# β-Hydroxybutyrate suppresses M1 macrophage polarization through β-hydroxybutyrylation of the STAT1 protein

**DOI:** 10.1038/s41419-024-07268-3

**Published:** 2024-12-03

**Authors:** Ya-Ping Bai, Yu-Jie Xing, Tao Ma, Kai Li, Teng Zhang, De-Guo Wang, Shu-Jun Wan, Cui-Wei Zhang, Yue Sun, Meng-Yan Wang, Guo-Dong Wang, Wen-Jun Pei, Kun Lv, Yan Zhang, Xiang Kong

**Affiliations:** 1 Anhui Provincial Key Laboratory of Non-coding RNA Basic and Clinical Transformation, Wannan Medical College, Wuhu, China; 2 College of Life Sciences, Anhui Normal University, Wuhu, China; 3 Department of Gastroenterology, The First Affiliated Hospital of Wannan Medical College, Wuhu, China; 4 Department of Gerontology, Geriatric Endocrinology unit, The First Affiliated Hospital of Wannan Medical College, Wuhu, China; 5 National Clinical Research Center for Geriatric Diseases, Anhui Provincial Sub-center, Wuhu, China; 6 School of Pharmacy, Wannan Medical College, Wuhu, China; 7 Anhui Province Key Laboratory of Biological Macro-molecules Research, Wannan Medical College, Wuhu, China

**Keywords:** Post-translational modifications, Post-translational modifications

## Abstract

β-Hydroxybutyrate (β-OHB), the primary ketone body, is a bioactive metabolite that acts as both an energy substrate and a signaling molecule. Recent studies found that β-OHB inhibits the production of pro-inflammatory cytokines in macrophages, but its underlying molecular mechanisms have not yet been fully elucidated. Lysine β-hydroxybutyrylation (Kbhb), a post-translational modification mediated by β-OHB, plays a key role in regulating the expression and activity of modified proteins. However, whether macrophages undergo protein Kbhb and whether Kbhb modification regulates macrophage polarization remains largely unknown. In this study, treatment with β-OHB and ketone ester significantly decreased the lipopolysaccharide (LPS)-induced enhancement of the M1 phenotype of mouse bone marrow-derived macrophages (BMDMs), RAW264.7 cells, and peritoneal macrophages (PMs) in vitro and in vivo. Moreover, β-OHB treatment induced global protein Kbhb, which is associated with the regulation of macrophage M1 polarization. Proteome-wide Kbhb analysis in β-OHB-treated BMDMs revealed 3469 Kbhb modification sites within 1549 proteins, among which interleukin-12-responding proteins were significantly upregulated. Our results indicated that β-OHB regulated M1 macrophage polarization by inducing Kbhb modification of the signal transducer and activator of transcription 1 (STAT1) K679 site, which inhibited its LPS-induced phosphorylation and transcription. Altogether, our study demonstrated the presence of a widespread Kbhb landscape in the β-OHB-treated macrophages and provided novel insights into the anti-inflammatory effects of β-OHB.

## Introduction

Macrophages are important immune cells that are characterized by plasticity, heterogeneity, and multipotency [1]. Under different micro-environments, macrophages polarize into two phenotypes, the classically-activated pro-inflammatory M1 phenotype and alternatively-activated reparative M2 phenotype, which play different roles in immune inflammatory responses and tissue repair [2, 3]. The M1 macrophages, induced by lipopolysaccharide (LPS) and/or interferon-gamma stimulation, are characterized by the upregulation of inducible nitric oxide synthase (iNOS) and overproduction of pro-inflammatory cytokines [4].

β-Hydroxybutyrate (β-OHB), the predominant ketone body, is produced in the liver and exhibits a wide range of bioactive properties [5]. β-OHB serves as an alternative energy source, as well as an important signaling molecule [6] and epigenetic regulator [7]. Moreover, β-OHB exhibits therapeutic effects in heart diseases and metabolic disorders [8–11]. Notably, β-OHB inhibits the activation of nucleotide-binding oligomerization domain-like receptor 3 inflammasome and nuclear factor kappa B in macrophages, reducing the production of pro-inflammatory cytokines [12, 13]. Ketone ester (KE, also known as BD-AcAc2), an exogenous ketone body compound, rapidly increases circulating β-OHB levels upon consumption [5]. Moreover, oral administration of KE decreases LPS-induced systemic inflammation in mice [14], indicating the anti-inflammatory effects of β-OHB. However, whether the anti-inflammatory effects of β-OHB are associated with the inhibition of M1 macrophage polarization remains unclear.

Post-translational modification (PTM) refers to the process in which different chemical functional groups are covalently added to some amino acid residues of a protein after translation, altering its structural and functional properties [15]. Lysine β-hydroxybutyrylation (Kbhb), a PTM mediated by β-OHB, plays a key role in regulating the expression and activity of modified histone and non-histone proteins [16, 17]. Starvation induces Kbhb in mice liver, which inhibits s-adenosyl-L-homocysteine hydrolase activity and facilitates adaptation to metabolic changes caused by energy deficiency [17]. Meanwhile, a ketogenic diet induces Kbhb of the hippocampal calcium/calmodulin-dependent protein kinase II alpha, which reduces cocaine reinstatement in addicted mice [18]. KE-induced Kbhb at the K395 site of citrate synthase promotes acetyl-CoA metabolism, reduces mitochondrial protein acetylation levels, and alleviates heart failure in mice [10]. However, whether macrophages undergo protein Kbhb and whether Kbhb modification regulates macrophage polarization remains largely unknown.

In this study, we found that β-OHB treatment inhibits M1 macrophage polarization, which is associated with the β-OHB-induced macrophage-specific Kbhb modification. Through proteome-wide Kbhb analysis, we identified 3469 upregulated Kbhb sites among 1549 proteins in the β-OHB-treated mouse bone marrow-derived macrophages (BMDMs). Furthermore, we demonstrated that β-OHB treatment significantly increased Kbhb modification of the signal transducer and activator of transcription 1 (STAT1) protein, subsequently reducing its phosphorylation and transcriptional activity, which are essential for M1 macrophage polarization.

## Methods

### Reagents and antibodies

β-OHB (#298360), LPS (#L2880), acetoacetate (AcAc, #54374), and phorbol 12-myristate 13-acetate (PMA, #P8139) were purchased from Sigma (Missouri, USA), and KE was purchased from DiBo Chemical Co. (#PB230618, Wuhan, China). The antibodies against iNOS (#A3774), Flag (#AE092), ubiquitin (#A19686), interleukin (IL)-6 (#A2447), and phosphorylated (P)-STAT1-S727 (#AP0453) were purchased from Abclonal (Wuhan, China). The antibodies against Kbhb (#PTM-1201RM), acetyllysine (Kac, #PTM-101), and lactyllysine (Kla, #PTM-1401RM) were purchased from PTM BioLabs (Hangzhou, China). The anti-β-hydroxybutyrate dehydrogenase 1 (BDH1, #BF0318) antibody was purchased from Affinity (Liyang, China), and the anti-STAT1 (#14994) and anti-P-STAT1-Y701 (#7649) antibodies were purchased from Cell Signaling Technology (Massachusetts, USA). The PE-conjugated anti-iNOS (#12-5920-82), PE-Cyanine7-conjugated anti-F4/80 (#25-4801-82), anti-IL-12 (#PA5-79460), and IgG (#10500C) antibodies were purchased from Thermo Fisher Scientific (Massachusetts, USA).

### Cell lines and culture conditions

The mouse RAW264.7 macrophage (#TIB-71) and L929 fibroblast (#CCL-1) cell lines were obtained from the American Type Culture Collection and have been used in our previous study [19]. The human THP-1 monocytic cell line (#CL-0233) was purchased from Procell (Wuhan, China). The HEK293T cell line (#CC4003) was purchased from Cellcook (Guangzhou, China). All the cell lines were verified and examined for mycoplasma contamination. The RAW264.7 and HEK293T cells were cultured in Dulbecco’s modified eagle medium (DMEM, #C11995500BT, Gibco, New York, USA) supplemented with 10% fetal bovine serum (FBS, #10099141C, Gibco). The THP-1 cells were cultured in RPMI-1640 medium (#C11875500BT, Gibco) containing 10% FBS and treated with 100 ng/ml PMA for 48 h to induce differentiation into macrophages in vitro [20].

The BMDMs were obtained as described in our previous study [19]. Briefly, bone marrow cells (BMCs) were isolated from the tibias and femurs of 9-week-old male C57BL/6 mice (GemPharmatech Co., Nanjing, China) and cultured for 7d in DMEM supplemented with 20% FBS, 20% L929 cell supernatant (LCS), and 1% penicillin/streptomycin (#C0222, Beyotime, Shanghai, China). Thereafter, the BMDMs were cultured for 24 h in fresh DMEM supplemented with 10% FBS. Subsequently, the BMDMs were subjected to experimental treatments.

Peritoneal macrophages (PMs) were obtained as previously described [21]. Briefly, the mice were sacrificed and intraperitoneally injected with 5 ml ice-cold phosphate-buffered saline (PBS). Thereafter, their peritoneal fluid was slowly extracted, centrifuged, re-suspended in RPMI-1640 medium supplemented with 10% FBS, and incubated for 4–6 h to allow the PMs to adhere. The non-adherent cells were washed away with PBS, and the isolated mouse PMs were used for subsequent experiments. The isolation of mouse Kupffer cells (KCs) was based on a two-step perfusion procedure [22] followed by density gradient centrifugation [23].

To explore the role of β-OHB in M1 macrophage polarization in vitro, the BMDMs and RAW264.7 cells were treated with 5 or 10 mM β-OHB for 24 h and subsequently treated with 100 ng/ml LPS for 24 h. The concentration of β-OHB was determined based on previous studies [12, 13], where 10 mM β-OHB was found to be sufficient for exerting anti-inflammatory effects in vitro.

To explore whether Kbhb modifications can persist and function in the absence of β-OHB, the RAW264.7 cells were cultured under β-OHB-free culture conditions for 24 and 48 h after inducing Kbhb modification by a 24-h β-OHB treatment. Furthermore, to confirm that Kbhb modification directly regulates M1 macrophage polarization, BMDMs were transfected with the recombinant adenovirus BDH1 (Ad-BDH1, Obio Technology Co., Ltd., Shanghai, China).

### Animal experiments

The experimental protocols conducted in this study were reviewed and approved by the Animal Ethics Committee of Wannan Medical College (approval number: WNMC-AWE-2024156, Anhui, China). The 8-week-old male C57BL/6 mice (GemPharmatech Co.) were acclimatized for 1 week and were housed in specific pathogen-free conditions under a 12/12-h light/dark cycle at 22–26 °C.

**Fig. 1:**
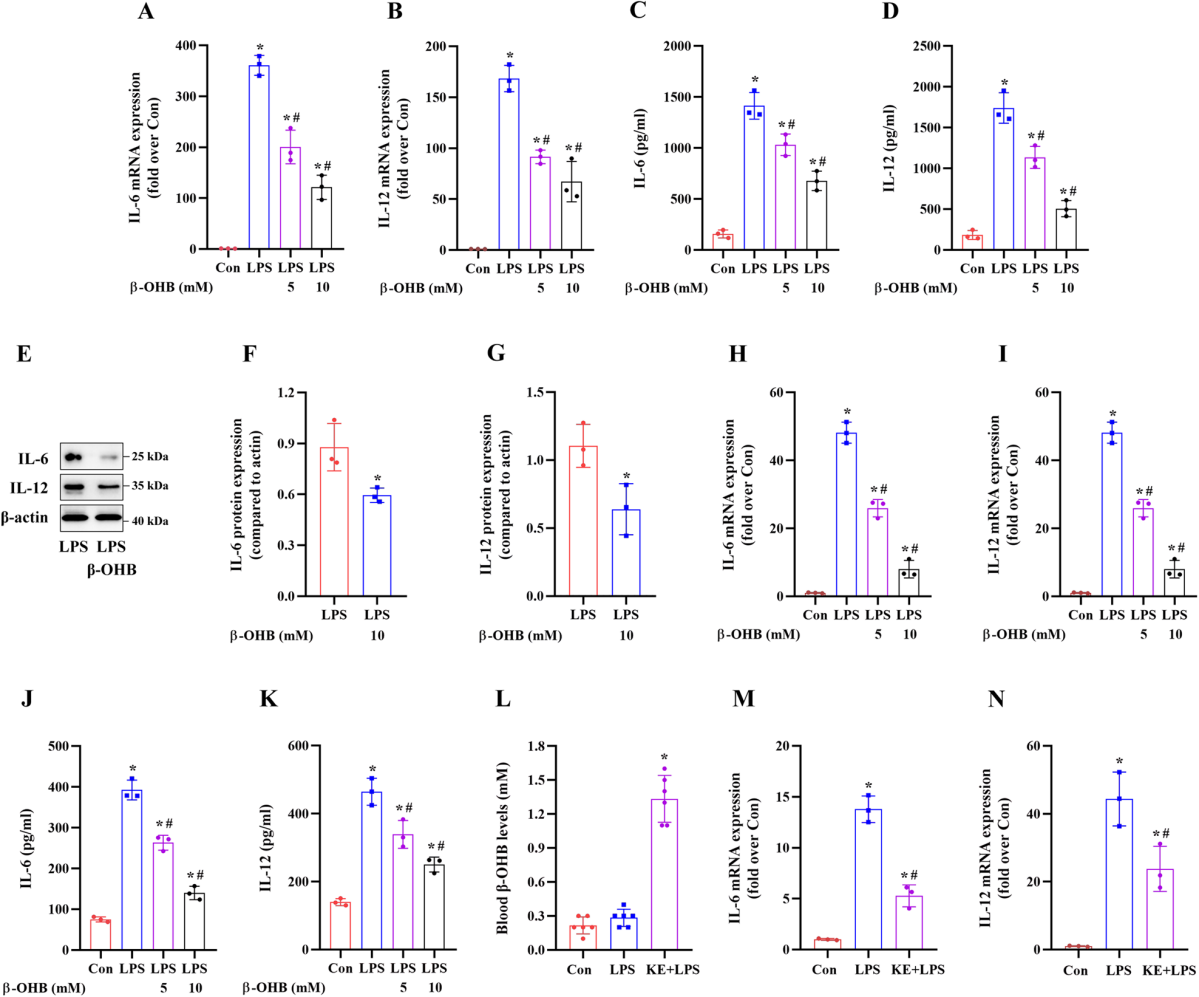
Effects of β-OHB treatment on macrophage IL-6 and IL-12 levels in vitro and in vivo. **A**–**G** BMDMs and **H**–**K** RAW264.7 cells were treated with 5 or 10 mM β-OHB for 24 h and then stimulated with 100 ng/ml LPS for 24 h. **A**, **B**, **H**, **I** RT-qPCR analysis of IL-6 and IL-12 mRNA expression levels in the BMDMs and RAW264.7 cells (n = 3/group). **C**, **D**, **J**, **K** ELISA was performed to measure the secretion of IL-6 and IL-12 in the BMDMs and RAW264.7 cells (n = 3/group). **E**–**G** The panel and histogram of IL-6 and IL-12 protein expression in the BMDMs (n = 3/group). The 9-week-old male C57BL/6 mice were orally administered 3 mg/g KE per day for 3d and then intraperitoneally injected with 5 mg/kg LPS and the PMs were collected for subsequent analysis. **L** The circulating β-OHB levels were measured (n = 6/group). **M**, **N** RT-qPCR analysis of IL-6 and IL-12 mRNA expression levels in the mouse PMs (n = 3/group). Data are presented as the mean ± standard deviation. *p < 0.05 vs Con group and ^#^*p* < 0.05 vs LPS group.

**Fig. 2:**
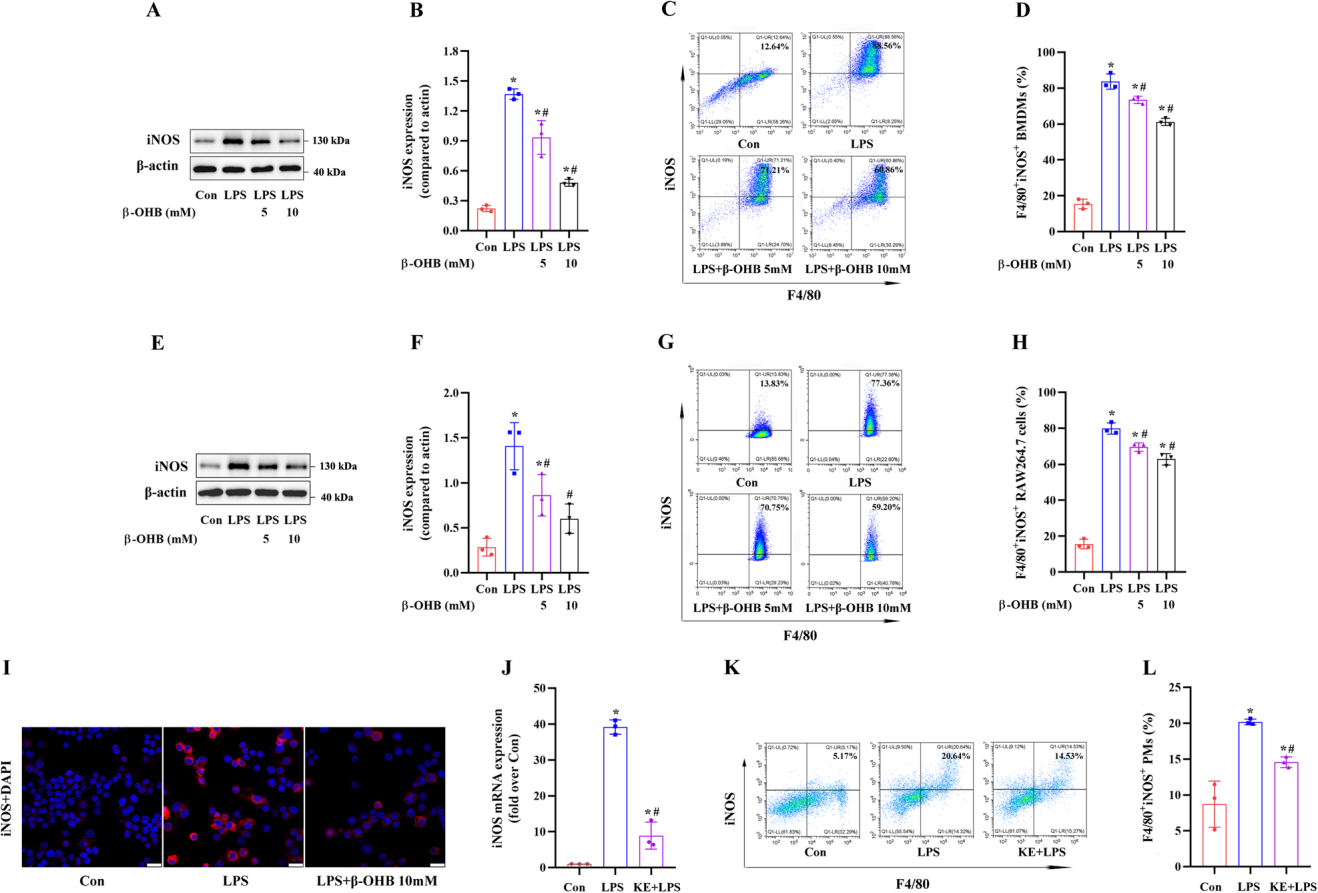
Effects of β-OHB treatment on M1 macrophage polarization in vitro and in vivo. **A**–**D** BMDMs and **E**–**I** RAW264.7 cells were treated with 5 or 10 mM β-OHB for 24 h and then treated with 100 ng/ml LPS for 24 h. **A**, **B**, **E**, **F** Immunoblotting analysis of iNOS protein expression in the BMDMs and RAW264.7 cells (n = 3/group). **C**, **D**, **G**, **H** FCM analysis of the proportion of M1 phenotype (iNOS^+^/F4/80^+^) BMDMs and RAW264.7 cells (n = 3/group). **I** Representative IF images of the iNOS protein in the RAW264.7 cells (scale bar = 25 μm). The 9-week-old male C57BL/6 mice were orally administered 3 mg/g KE per day for 3d and then intraperitoneally injected with 5 mg/kg LPS and the PMs were collected for subsequent analysis. **J** RT-qPCR analysis of iNOS mRNA expression levels in the mouse PMs (n = 3/group). **K**, **L** FCM analysis of the proportion of M1 phenotype (iNOS^+^/F4/80^+^) mouse PMs (n = 3/group). Data are presented as the mean ± standard deviation. *p < 0.05 vs Con group and ^#^*p* < 0.05 vs LPS group.

To explore the role of KE in M1 macrophage polarization in vivo, the mice were randomly divided into three groups of six mice each, as follows: control mice (Con), LPS-treated mice (LPS), and KE + LPS-treated mice (KE + LPS). The PMs were then isolated from these mice and used for subsequent experiments as shown in Figs. 1L–N and 2J–L.

**Fig. 3:**
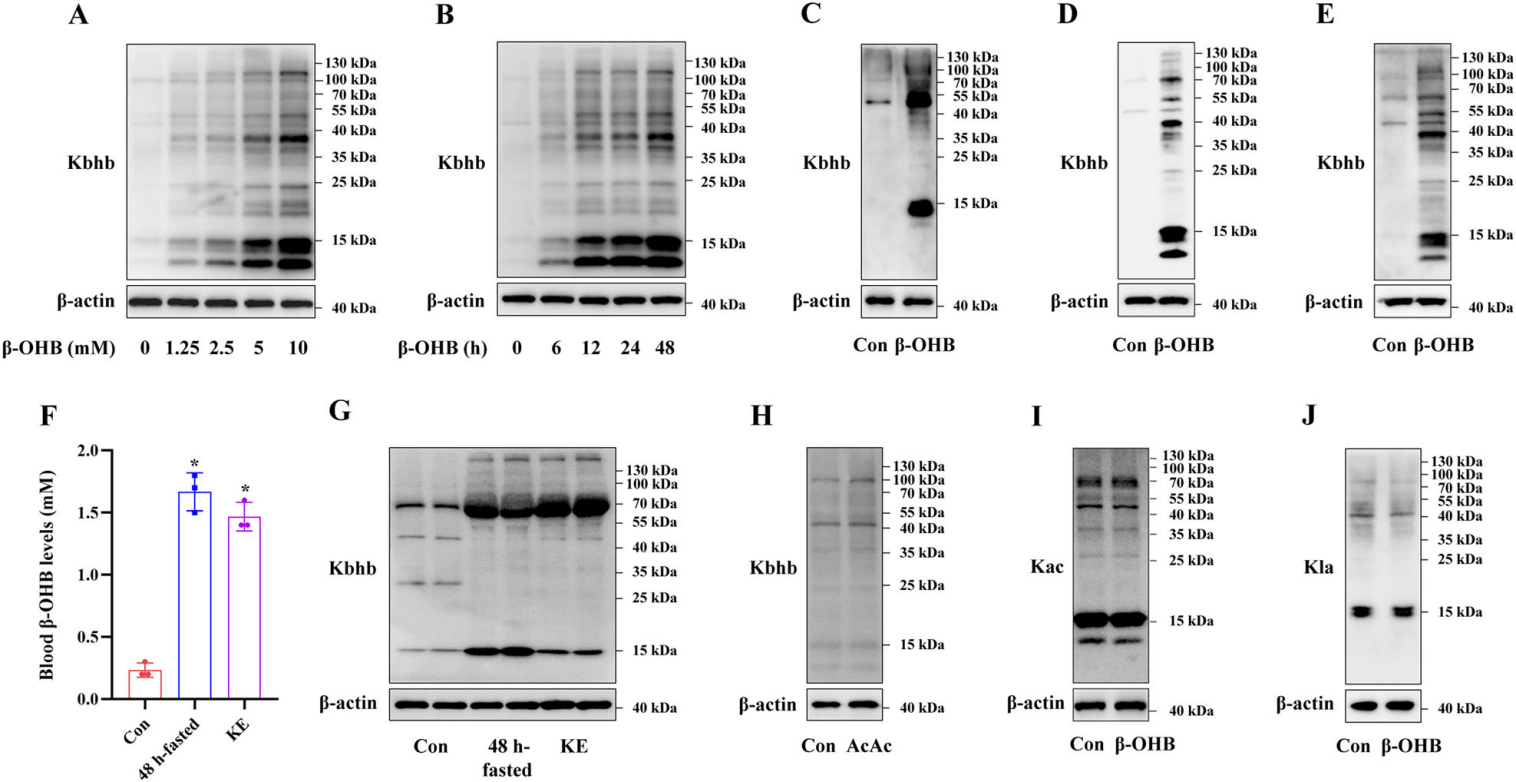
Effects of β-OHB treatment on macrophage-specific Kbhb modification. **A** RAW264.7 cells were treated with β-OHB at the indicated concentrations for 24 h (n = 3/group). **B** RAW264.7 cells were treated with 10 mM β-OHB at the indicated times (n = 3/group). **C** BMDMs were treated with 10 mM β-OHB for 24 h (n = 3/group). **D** THP-1 cells were differentiated into macrophages using PMA and then treated with 10 mM β-OHB for 24 h (n = 3/group). **E** KCs were treated with 10 mM β-OHB for 24 h (n = 3/group). Immunoblotting analysis of the Kbhb modification in the RAW264.7 cells and BMDMs. The 9-week-old male C57BL/6 mice were starved for 48 h or orally administered 3 mg/g KE per day for 3d. **F** The circulating β-OHB levels were measured. **G** Immunoblotting analysis of Kbhb modification in the mouse PMs (n = 3/group). **H** RAW264.7 cells were treated with 10 mM AcAc for 24 h (n = 3/group). **I**, **J** RAW264.7 cells were treated with 10 mM β-OHB for 24 h (n = 3/group). Immunoblotting analysis of the Kbhb, Kac, and Kla PTMs in the AcAc- and β-OHB-treated RAW264.7 cells. Data are presented as the mean ± standard deviation. *p < 0.05 vs Con group.

To detect macrophage Kbhb modification in vivo, the mice were randomly divided into three groups of three mice each, as follows: Con mice, 48-h fasted mice, and KE-treated mice. The PMs were then isolated from these mice and used for subsequent experiments as shown in Fig. 3D, E.

**Fig. 4:**
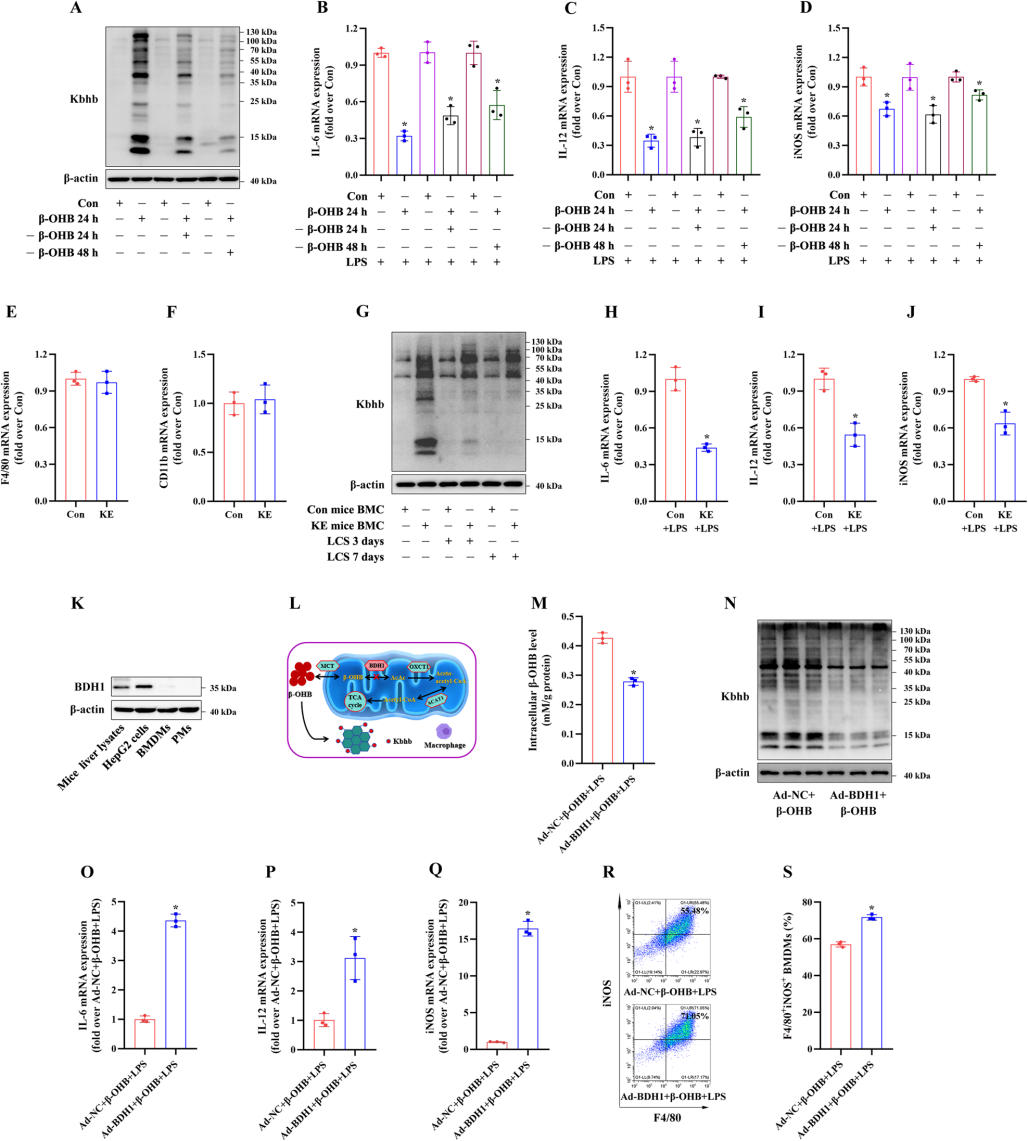
Effects of β-OHB treatment on the regulation of M1 macrophage polarization. RAW264.7 cells were first treated with 10 mM β-OHB for 24 h, then cultured under β-OHB-free culture conditions for 24 and 48 h, and stimulated with 100 ng/ml LPS for 24 h. **A** Immunoblotting analysis of the Kbhb modification in the RAW264.7 cells (n = 3/group). **B**–**D** RT-qPCR analysis of IL-6, IL-12, and iNOS mRNA expression levels in the RAW264.7 cells (n = 3/group). The 9-week-old male C57BL/6 mice were orally administered 3 mg/g KE per day for 3d, and their BMCs were obtained and cultured with LCS for 7d to induce BMC differentiation into BMDMs. **E**, **F** RT-qPCR analysis of F4/80 and CD11b mRNA expression levels in the BMDMs (n = 3/group). **G** Immunoblotting analysis of the Kbhb modification in the BMCs treated with LCS at the indicated times (n = 3/group). Partial BMDMs were stimulated with 100 ng/ml LPS for 24 h. **H**–**J** RT-qPCR analysis of IL-6, IL-12, and iNOS mRNA expression levels in the BMDMs (n = 3/group). **K** BDH1 protein expression in mice liver lysates, HepG2 cells, BMDMs, and PMs. **L** Lack of BDH1 expression leads to β-OHB accumulation in macrophages, which may induce its Kbhb modification. BMDMs were transfected with either Ad-normal control (NC) or Ad-BDH1, then treated with 10 mM β-OHB for 24 h, followed by stimulation with 100 ng/ml LPS for an additional 24 h. **M** The intracellular β-OHB levels were measured (n = 3/group). **N** Immunoblotting analysis of the Kbhb modification in the BMDMs (n = 3/group). **O**–**Q** RT-qPCR analysis of IL-6, IL-12, and iNOS mRNA expression levels in the BMDMs (n = 3/group). **R**, **S** FCM analysis of the proportion of M1 phenotype (iNOS^+^/F4/80^+^) mouse BMDMs (n = 3/group). Data are presented as the mean ± standard deviation. *p < 0.05 vs Con, Con + LPS, or Ad-NC + β-OHB + LPS group.

To explore whether Kbhb modifications can persist and function continuously in vivo, the mice were randomly divided into two groups of three mice each, as follows: Con mice and KE-treated mice. The BMCs were then isolated from these mice and used for subsequent experiments as shown in Fig. 4E–J.

To explore the effect of STAT1 Kbhb in M1 macrophage polarization in vivo, the mice were randomly divided into two groups of six mice each. The Group 1 mice were injected with an adeno-associated virus serotype 2/9 (AAV2/9) vector carrying the mouse STAT1 gene (AAV2/9-m-STAT1-Flag), while the Group 2 mice were injected with an AAV2/9 vector carrying the mouse mutant STAT1 gene (AAV2/9-m-STAT1-K679R-Flag). All the AAV2/9 vectors were manufactured by HanBio Biotechnology Co., Ltd. (Shanghai, China). The mice were injected in each femur with 30 μl of virus containing 1 × 10^13^ AAV2/9 vector genomes [24, 25]. The mice were then subjected to the KE + LPS treatment after 2 weeks of transfection. The PMs were then isolated from these mice and used for subsequent experiments as shown in Fig. 10.

The mice were administered 3 mg/g/d KE through intragastric gavage at 10 AM for three consecutive days. The dosage of KE was determined based on previous studies [14, 26], which found that 3 mg/g/d KE significantly increases circulating β-OHB levels without causing ketoacidosis. Thereafter, 5 mg/kg LPS was administered to the mice through intraperitoneal injection [26]. No deaths were recorded within 24 h after LPS administration.

### Biochemical analysis and enzyme-linked immunosorbent assay (ELISA)

The β-OHB levels in mice tail blood samples were measured using a ketone meter (#FreeStyle Optium Neo, Abbott, Oxon, UK). The intracellular β-OHB levels were measured using a colorimetric assay kit (#700190, Cayman Chemical, Michigan, USA), according to the manufacturer’s instructions. The IL-6 and IL-12 levels in macrophage supernatants were measured using cytokine ELISA kits (#E-EL-M0044 and E-EL-M3062, Elabscience, Wuhan, China), according to the manufacturer’s instructions.

### Immunoblotting analysis

Total proteins from the macrophages and HEK293T cells were isolated and separated through sodium dodecyl sulfate–polyacrylamide gel electrophoresis and transferred onto polyvinylidene fluoride membranes [27–29]. The membranes were incubated overnight with anti-iNOS (1:1000), IL-6 (1:1000), IL-12 (1:1000), Flag (1:2000), ubiquitin (1:1000), Kbhb (1:1000), Kac (1:1000), Kla (1:1000), BDH1 (1:1000), P-STAT1-S727 (1:1000), P-STAT1-Y701 (1:500), and STAT1 (1:1000) primary antibodies. Subsequently, the membranes were incubated with the corresponding secondary antibodies for 2 h. The antibody-bound proteins were detected using an enhanced chemiluminescence kit (#MK042A, BIOMIKY, Shanghai, China), and the data was analyzed using the ImageJ software.

### Cellular immunofluorescence (IF) staining

The RAW264.7 cells were washed and fixed for permeabilization. Thereafter, the cells were blocked, incubated with anti‐iNOS antibodies (1:200) for 2 h at room temperature, and then incubated with goat anti-rabbit recombinant secondary antibodies (#SA00013-4, 1:100, Proteintech, Wuhan, China) for 2 h. Subsequently, the cells were stained with 4’,6-diamidino-2-phenylindole (#BL105A, Biosharp, Hefei, China) for 10 min for nuclear staining. Lastly, the cells were observed under the Zeiss LSM 780 confocal microscope (Oberkochen, Germany).

### Flow cytometry (FCM) analysis

To determine the proportion of the M1 macrophages (iNOS+/F4/80+ cell), the RAW264.7 cells, BMDMs, and PMs were first incubated with PE-Cyanine7-conjugated anti-F4/80 antibodies for 15 min in the dark at room temperature and then fixed for 15 min at room temperature with intracellular fixation buffer (#00-8222-49, Thermo Fisher Scientific). Thereafter, the cells were permeabilized with membrane disruption buffer (#00-8333-56, Thermo Fisher Scientific) at 4 °C for 45 min and then incubated with PE-conjugated anti-iNOS antibodies for 50 min at room temperature. Subsequently, the cells were washed thrice and analyzed using the Cytomics FC500 MPL flow cytometer (Beckman, California, USA). The data were analyzed using the FlowJo software.

### Reverse transcription-quantitative polymerase chain reaction (RT-qPCR) analysis

Total RNA was extracted from the macrophages using TRIzol reagent (#15596026N, Invitrogen, California, USA), and 1.5 mg RNA was used to synthesize complementary DNA using a first strand cDNA synthesis kit (#BL696A, Biosharp). RT-qPCR analysis was performed on the CFX Connect fluorescent qPCR instrument (Bio-Rad Laboratories, Inc., California, USA) using the SYBR Green Master Mix (#BL697A, Biosharp). The expression levels of the target genes were calculated using the 2^−ΔΔCt^ method and normalized to those of β-actin. The primers (Sangon Biotech, Shanghai, China) used for the RT-qPCR analysis are listed in Table 1.

**Table 1:** The primer sequences used for the RT-qPCR analysis.

Genes	Primers	Primer sequences (5′→3′)
IL-6	Forward	AGTTGCCTTCTTGGGACTGA
	Reverse	TCCACGATTTCCCAGAGAAC
IL-12	Forward	GACCTGTTTACCACTGGAACTA
	Reverse	GATCTGCTGATGGTTGTGATTC
TNF-α	Forward	CCCACGTCGTAGCAAACCA
	Reverse	ACAAGGTACAACCCATCGGC
IL-1β	Forward	TGCCACCTTTTGACAGTGATG
	Reverse	AAGGTCCACGGGAAAGACAC
iNOS	Forward	GTTCTCAGCCCAACAATACAAGA
	Reverse	GTGGACGGGTCGATGTCAC
CD11b	Forward	CAGATCAACAATGTGACCGTATGGG
	Reverse	CATCATGTCCTTGTACTGCCGCTTG
F4/80	Forward	CTGCACCTGTAAACGAGGCTT
	Reverse	GCAGACTGAGTTAGGACCACAA
β-actin	Forward	GTGACGTTGACATCCGTAAAGA
	Reverse	GCCGGACTCATCGTACTCC

### Global Kbhb proteomic analysis

The BMDMs were incubated in the presence or absence of 10 mM β-OHB for 24 h and then subjected to 4-dimensional label-free Kbhb proteomics conducted by PTM BioLabs. Briefly, the extracted BMDM proteins were digested with trypsin, and the fractionated samples were incubated with anti-Kbhb beads (#PTM-1204, PTM BioLabs) to enrich Kbhb-modified peptides [18]. The peptides were analyzed using an NSI source, followed by tandem mass spectrometry in the Q ExactiveTM Plus system, which was coupled with UPLC. The mass spectrometry (MS) data were processed using the Maxquant search engine (v1.6.6.0). The subcellular localization of modified proteins was predicted using the WolF PSORT software. Lastly, functional enrichment of the modified proteins was conducted using Gene Ontology (GO) analysis.

### Generation of *STAT1*-knockout (KO) RAW264.7 cells

The STAT1-KO RAW264.7 cells were manufactured by Obio Technology Co., Ltd. using CRISPR‒Cas9 gene editing technology. Briefly, the guide RNA (5′-GGTCGCAAACGAGACATCAT-3′) was cloned into the lentivirus–cas9 vector and the vector was then transfected into the RAW264.7 cells. Subsequently, the KO cell lines were selected for further analysis. The selected STAT1-KO cell lines were verified by immunoblotting and Sanger sequencing analyses.

### Construction of mutant plasmids

The pCMV-3×Flag-Stat1 (mouse)-Neo plasmids (#P44615) were obtained from MiaoLingBio (Wuhan, China). The site-directed mutation plasmids of STAT1 (K193R, K286R, K336R, K379R, K652R, K679R, and K679T) were generated from the wild-type (WT) Flag-STAT1 plasmids using the KOD-Plus-Mutagenesis kit (#SMK101, TOYOBO, Tokyo, Japan). The generated mutant plasmids were validated through Sanger sequencing and aligned with the reference sequence (RefSeq accession number NM_001357627). The primers used to generate the site-directed mutation plasmids are listed in Table 2. The HEK293T and STAT1-KO RAW264.7 cells were transfected with the mutant plasmids using the GenMute™ siRNA&DNA Transfection reagent (#SL100568, SignaGen, Gaithersburg, USA) and GenMute™ Transfection buffer (#SL100572, SignaGen).

**Table 2:** The primer sequences used for generating STAT1 site-directed mutation plasmids.

Genes	Primers	Primer sequences (5′→3′)
STAT1-K193R	Forward	AGACAGGAACAGCTGCTGCTCCACAAGAT
	Reverse	TTGGTCGCTCTTCGCCACACCATTGGCT
STAT1-K286R	Forward	AGATTCACCTATGAGCCCGACCCTATT
	Reverse	CTGTTCCAACTCCTCCAGCTTTTTAAGCT
STAT1-K336R	Forward	AGAACTGGGGTACAGTTCACTGTCAAGCT
	Reverse	CAAGACCAGGGGCCTCTGCGGGT
STAT1-K379R	Forward	AGGTTCAACATCTTGGGTACGCACACAAAAGT
	Reverse	CCGAAATCCTTTAACTGTGTTTTTCTCGT
STAT1-K652R	Forward	TACAGAGTCATGGCTGCCGAGAACAT
	Reverse	GTTGCGAATAATATCTGGGAAAGT
STAT1-K679R	Forward	AGATATTATTCCAGACCAAAGGAAGCACC
	Reverse	CCCAAAGGCGTGGTCTTTGTCAAT
STAT1-K679T	Forward	ACGTATTATTCCAGACCAAAGGAAGCACC
	Reverse	CCCAAAGGCGTGGTCTTTGTCAAT

### Co-immunoprecipitation (Co-IP) analysis

Endogenous Co-IP assays were performed using the Pierce™ Classic Magnetic IP/Co-IP kit (#88804, Thermo Fisher Scientific) with anti-STAT1 and IgG (control) antibodies, while exogenous Co-IP assays were conducted using anti-Flag magnetic beads (#HY-K0207, MedChemExpress, Shanghai, China). The bound proteins were eluted, and the supernatants were collected for immunoblotting analysis of Kbhb, ubiquitin, and Kac.

### Dual-luciferase reporter assay

The HEK293T cells were transfected with Flag-STAT1-WT, -K286R, -K679R, or -K679T plasmids; pGL6-STAT1-TA-Luc plasmid (#D4400, Beyotime); and Renilla luciferase plasmid (#D2760, Beyotime). The dual-luciferase reporter assay was performed on a GLOMAX® Multi-Mode Microplate Reader (Promega, Wisconsin, USA) using a dual-luciferase assay kit (#E1910, Promega). The activity of Renilla luciferase was normalized to that of the Firefly luciferase [2].

### Statistical analysis

Data are presented as the mean ± standard deviation of three independent experiments. Differences between multiple groups were analyzed by one-way analysis of variance, followed by Tukey’s multiple comparison test, while differences between two groups were compared by a two-tailed Student’s t-test. The *P*-value < 0.05 was considered to be statistically significant.

## Results

### β-OHB inhibited M1 macrophage polarization in vitro and in vivo

Previous studies indicated that β-OHB reduced pro-inflammatory cytokines production [12–14, 30, 31]. In the preliminary experiments of the present study, 24-h treatment with β-OHB reduced the LPS-induced increase in the mRNA expression of IL-6, IL-12, TNF-α, and IL-1β (Fig. S1A). Similar to previous reports [12, 30], among these reduced pro-inflammatory cytokines, β-OHB had a stronger effect on IL-6 and IL-12 expression. Therefore, in subsequent studies, we used IL-6 and IL-12 as markers to evaluate the effects of β-OHB on M1 macrophage polarization.

Consistently, our results revealed that 24-h treatment with β-OHB significantly decreased the LPS-induced increase in IL-6 and IL-12 generation and secretion in BMDMs (Fig. 1A–G) and RAW264.7 cells (Fig. 1H–K). Intraperitoneal injection of LPS significantly upregulated IL-6 and IL-12 mRNA expression in the mouse PMs; however, KE administration for 3d significantly increased the circulating β-OHB levels (Fig. 1L) and downregulated the LPS-induced IL-6 and IL-12 expression in mouse PMs (Fig. 1M, N). These findings further confirmed that β-OHB exhibited anti-inflammatory effects in vitro and in vivo.

To explore the potential anti-inflammatory mechanisms of β-OHB, we conducted proteomics analysis to identify differentially expressed proteins (DEPs) in the control and β-OHB-treated BMDMs. Based on the expression profiling data and the set threshold values (adjusted P-value ≤ 0.05 and |log2 (fold change)| ≥1.5), we identified 51 upregulated and 59 downregulated DEPs in the β-OHB-incubated BMDMs compared to the control BMDMs (Fig. S1B). The M1 macrophage markers, such as cluster of differentiation 74, perilipins 2, and prostaglandin-endoperoxide synthase [32–34], were downregulated in the β-OHB-incubated BMDMs (Fig. S1C).

To further clarify the effects of β-OHB on macrophage polarization, we used LPS-induced in vitro and in vivo M1 macrophage polarization models. The results revealed that β-OHB treatment significantly decreased the LPS-induced iNOS (M1 marker) protein expression and partially attenuated the LPS-induced increase in M1 phenotype (iNOS^+^/F4/80^+^) macrophages in BMDMs (Fig. 2A–D) and RAW264.7 cells (Fig. 2E–H). The results of IF staining of iNOS in RAW264.7 cells were consistent with these findings (Fig. 2I). Intraperitoneal injection of LPS upregulated iNOS mRNA expression in PMs and enhanced the number of M1 PMs in vivo; however, KE administration significantly suppressed these LPS-induced effects in PMs (Fig. 2J–L). These results demonstrated that β-OHB treatment inhibited M1 macrophage polarization, contributing to a decrease in the production of pro-inflammatory cytokines in macrophages.

### β-OHB-mediated macrophage-specific Kbhb modification in vitro and in vivo

β-OHB serves as a β-hydroxybutyryl donor for PTM Kbhb modification [16]. To explore whether β-OHB can induce global protein Kbhb modification in macrophages, we conducted immunoblotting assays using a Kbhb antibody. As shown in Fig. 3A, B, β-OHB treatment induced a significant dose- and time-dependent increase in Kbhb modification in RAW264.7 cells. Similarly, β-OHB treatment increased Kbhb modification in BMDMs (Fig. 3C), PMA differentiated-THP-1 macrophages (Fig. 3D), and KCs (Fig. 3E). Furthermore, both 48-h starvation and 3-d KE administration elevated circulating β-OHB levels in mice (Fig. 3F) and increased Kbhb modification in mouse PMs (Fig. 3G). Notably, AcAc did not induce Kbhb modification in macrophages (Fig. 3H), while β-OHB did not induce Kac and Kla PTMs in RAW264.7 cells (Fig. 3I, J). These findings indicated that β-OHB was associated with global protein Kbhb modification in macrophages, both in vitro and in vivo.

### β-OHB regulated M1 macrophage polarization through Kbhb modification

As shown in Fig. 4A–D, after 24 h of BHB treatment, RAW264.7 cells continued to be cultured under β-OHB-free conditions. Kbhb modification persisted for 24–48 h and continued to inhibit its IL-6, IL-12, and iNOS mRNA expression.

The BMCs were cultured with LCS for 7d to differentiate into BMDM in vitro. Our results revealed that KE treatment did not affect the BMDM differentiation, similar to a previous study, which found that β-OHB does not impact the differentiation of THP-1 cells into macrophages [35]. This was because differentiated BMDMs from the BMCs of control and KE-treated mice exhibited no significant difference in the expression of macrophage markers F4/80 and CD11b (Fig. 4E, F).

During the differentiation of BMC into BMDM, cells were cultured under conditions free of β-OHB. Notably, Kbhb modification persisted at both 3 and 7d during BMDM differentiation (Fig. 4G). Moreover, the differentiated BMDMs from the BMCs of the KE-treated mice continued to show the inhibition of IL-6, IL-12, and iNOS mRNA expression compared to those obtained from the BMCs of the control mice (Fig. 4H–J).

BDH1, a key rate-limiting enzyme in ketone body metabolism, regulates the interconversion between β-OHB and AcAc [5]. AcAc is converted to acetoacetyl-CoA by 3-oxoacid CoA transferase 1, which is further converted to acetyl-CoA by acetoacetyl-CoA thiolase for entry into the tricarboxylic acid cycle. Consistent with a previous study [36], our results showed no observable BDH1 protein expression in BMDMs and PMs (Fig. 4K), which may lead to β-OHB accumulation in macrophages and subsequently lead to the induction of Kbhb modification (Fig. 4L).

To further clarify the effects of Kbhb modification on macrophage polarization, we upregulated BDH1 expression in BMDMs using Ad-BDH1. The overexpression efficiency of Ad-BDH1 is shown in Fig. S2A. Overexpression of BDH1 did not affect the inflammatory phenotype of BMDMs (Fig. S2B–D). However, in BMDMs incubated with β-OHB, BDH1 overexpression significantly reduced the intracellular β-OHB content (Fig. 4M) and the level of Kbhb modifications (Fig. 4N), and subsequently reduced the inhibitory effects of β-OHB on macrophage M1 polarization (Fig. 4O–S).

### Kbhb-modified proteins were enriched in IL-12 response and Fc-gamma receptor signaling

**Fig. 5:**
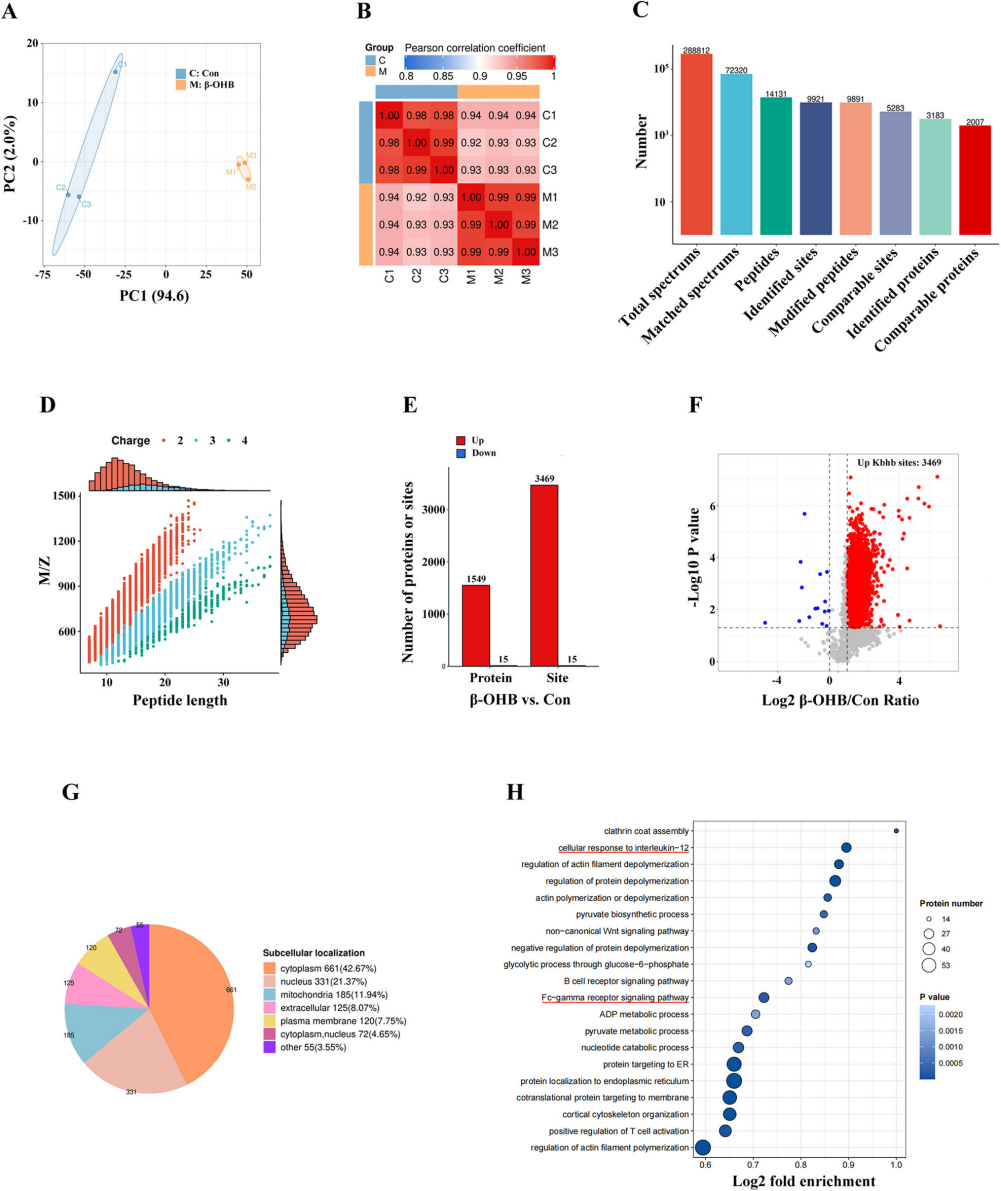
Global proteomic analysis of the β-OHB-induced Kbhb modification in mouse BMDMs. **A** Two-dimensional scatterplot of principal component analysis. **B** Heat map of Pearson’s correlation coefficient. **C** Overview of protein identification. **D** Length distribution of all identified peptides. **E** Histogram of the differential proteins and Kbhb sites in the β-OHB-treated and control BMDMs. **F** Volcano plot of the significantly differential Kbhb sites in the β-OHB-treated and control BMDMs. **G** Subcellular localization of the differential Kbhb-modified proteins. **H** GO functional enrichment analysis of the proteins with upregulated Kbhb sites in the biological processes category.

To determine the effects of Kbhb modification on the regulation of macrophage M1 polarization, we conducted a proteomic analysis of the global protein Kbhb in the β-OHB-treated BMDMs. Principal component analysis (Fig. 5A) and Pearson’s correlation coefficient analysis (Fig. 5B) were used to evaluate sample repeatability, and the findings indicated strong aggregation and quantitative consistency. A total of 288,812 spectra were detected, with 72,320 matched spectra and 14,131 peptides identified. Overall, 3183 proteins and 9921 Kbhb sites were recognized (Fig. 5C). The majority of the peptides were 7–20 amino acids long, consistent with the standard principles of enzymatic hydrolysis and MS fragmentation (Fig. 5D). According to the expression profiling data and the specified threshold values (adjusted P-value ≤ 0.05 and |log2 (fold change)| ≥1.5), we identified 3,469 upregulated differential Kbhb sites from 1549 proteins and 15 downregulated differential Kbhb sites from 15 proteins in the β-OHB-incubated BMDMs compared to the control BMDMs (Fig. 5E, F). Cellular localization analysis indicated that the Kbhb-modified proteins were primarily located in the cytoplasm, nucleus, and mitochondria (Fig. 5G). GO enrichment analysis revealed that the Kbhb-modified proteins were enriched in IL-12 response and Fc-gamma receptor signaling (Fig. S3A and Fig. 5H), which are crucial for M1 macrophage programming [34, 37].

### β-OHB promoted Kbhb modification of the STAT1 K679 site

**Fig. 6:**
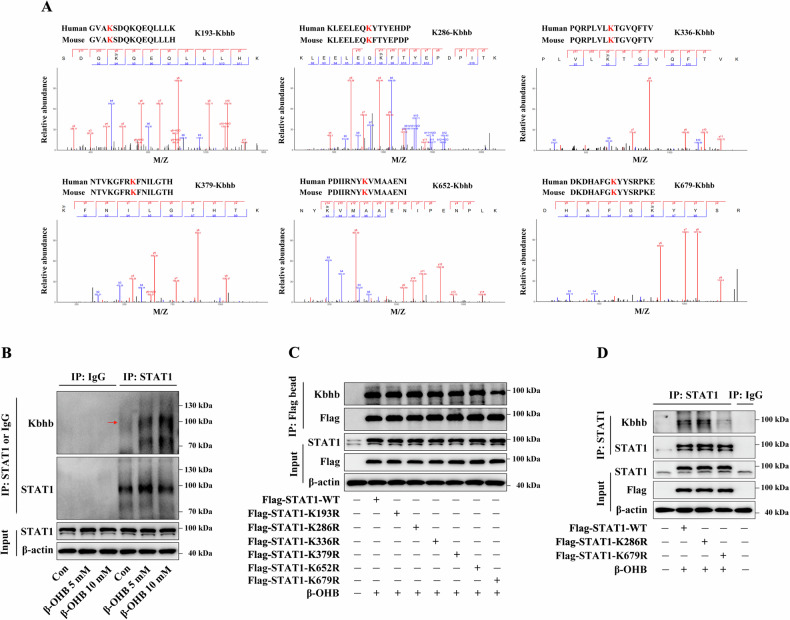
Effects of β-OHB treatment on STAT1 Kbhb modification. **A** The MS data of STAT1 Kbhb sites (K193, K286, K336, K379, K652, and K679) identified in the β-OHB-treated BMDMs. Top: conservation of Kbhb sites in mouse and human STAT1 proteins. **B** RAW264.7 cells were treated with or without 5 or 10 mM β-OHB for 24 h. Immunoblotting analysis of STAT1 Kbhb levels in RAW264.7 cells. **C**, **D** HEK293T cells were transfected with the indicated plasmids and treated with 10 mM β-OHB for 24 h. Immunoblotting analysis of STAT1 Kbhb levels in HEK293T cells. Immunoblotting data represents the results of three independent experiments.

STAT1, a key signaling molecule involved in the regulation of M1 macrophage polarization [38–40], was enriched among the Kbhb-modified proteins on the IL-12 response signaling (Fig. S3B). The MS data revealed an obvious mass shift at six sites (K193, K286, K336, K379, K652, and K679) of the STAT1 protein in the β-OHB-treated BMDMs, indicating that these sites were significantly β-hydroxybutyrylated (Fig. 6A). Furthermore, sequence analysis of the STAT1 protein revealed the presence of homologous sequences near these amino acid residues in both humans and mice, indicating that the Kbhb sites of the STAT1 protein are conserved (Fig. 6A). Immunoblotting analysis showed that β-OHB treatment significantly elevated Kbhb levels of STAT1 protein in RAW264.7 cells, consistent with the results of MS analysis (Fig. 6B).

The HEK293T cells were transfected with Flag-STAT1-WT or -mutant (lysine residues mutated to arginine at six Kbhb sites) plasmids and immunoprecipitated using anti-Flag beads (Fig. 6C) or anti-STAT1 antibodies (Fig. 6D). The results revealed that the STAT1-K679R mutant showed significantly reduced Kbhb modification levels compared to the WT or other STAT1 mutants, suggesting that the K679 site was the primary Kbhb site of STAT1.

### Kbhb modification of the STAT1 K679 site inhibited M1 macrophage polarization in vitro

The STAT1-KO RAW264.7 cells were constructed using CRISPR/Cas9 gene editing technology to study the effect of Kbhb modification on M1 macrophage polarization in the absence of endogenous STAT1 (Fig. S4A, B). Compared to normal RAW264.7 cells (Fig. 2G, H), the LPS-induced increase in the M1 phenotype was significantly reduced in STAT1-KO RAW264.7 cells (Fig. S4C, D), proving that STAT1 is an important regulatory factor for macrophage M1 polarization.

**Fig. 7:**
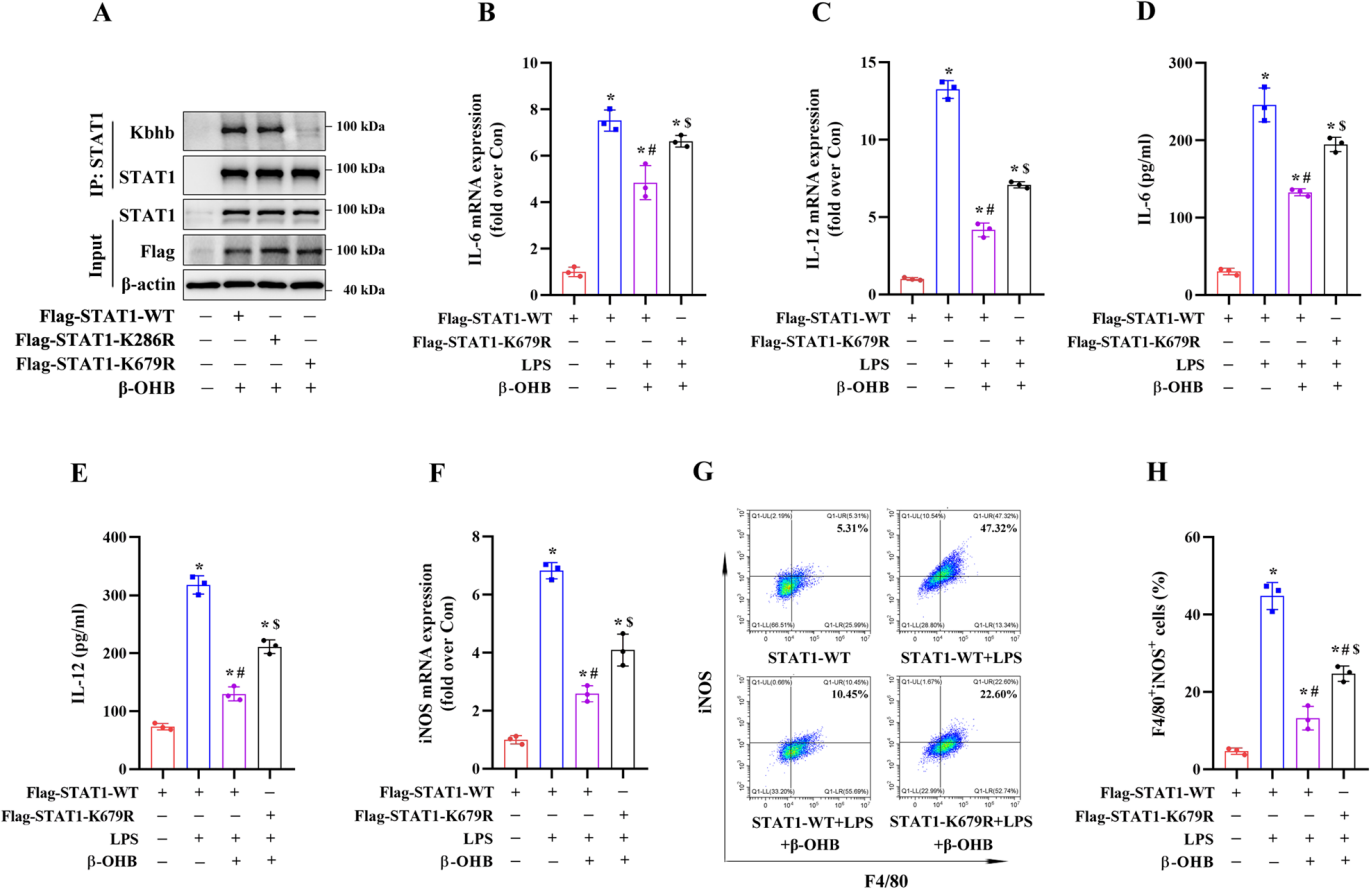
Effects of STAT1-K679R mutant on macrophage M1 polarization. STAT1-KO RAW264.7 cells were transfected with the indicated plasmids, treated with 10 mM β-OHB for 24 h, and stimulated with 100 ng/ml LPS for 24 h. **A** Immunoblotting analysis of STAT1 Kbhb levels. **B**, **C** RT-qPCR analysis of IL-6 and IL-12 mRNA expression levels. **D**, **E** ELISA was performed to measure the secretion of IL-6 and IL-12. **F** RT-qPCR analysis of iNOS mRNA expression levels. **G**, **H** FCM analysis of the proportion of M1 phenotype (iNOS^+^/F4/80^+^). Data are presented as the mean ± standard deviation of three independent experiments. *p < 0.05 vs Flag-STAT1-WT group, ^#^*p* < 0.05 vs Flag-STAT1-WT + LPS group, and ^$^*p* < 0.05 vs Flag-STAT1^-^WT + LPS + β-OHB group.

Transfection with the STAT1-K679R plasmids decreased the β-OHB-induced STAT1 Kbhb levels in the STAT1-KO RAW264.7 cells compared to transfection with the STAT1-WT or STAT1-K286R plasmids (Fig. 7A). This was consistent with the results obtained from HEK293T cells and further confirmed that the 679 site is an important KBHB modification site for the STAT1 protein. LPS stimulation significantly increased IL-6 and IL-12 mRNA expression and secretion, upregulated iNOS mRNA expression, and enhanced the ratio of M1 macrophages in STAT1-KO RAW264.7 cells transfected with STAT1-WT plasmids (Fig. 7B–H). Treatment with β-OHB significantly suppressed these LPS-induced effects in STAT1-KO RAW264.7 cells transfected with STAT1-WT plasmids (Fig. 7B–H). Moreover, compared to transfection with the STAT1-WT plasmids, transfection with the STAT1-K679R plasmids significantly enhanced these LPS-induced effects in β-OHB-treated STAT1-KO RAW264.7 cells (Fig. 7B–H). These results suggested that Kbhb modification of the STAT1 K679 site reduced the inhibitory effects of β-OHB on macrophage M1 polarization.

**Fig. 8:**
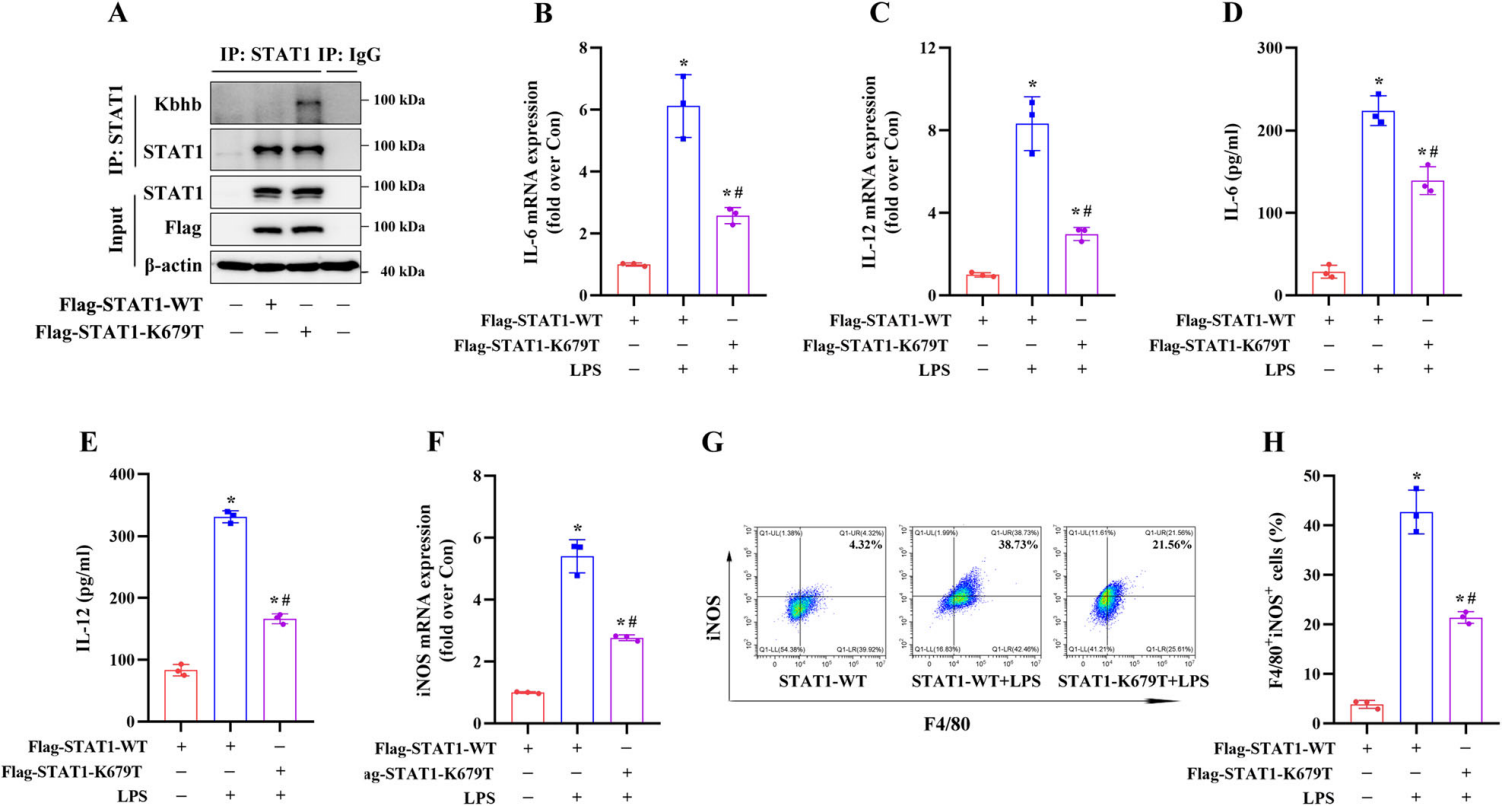
Effects of STAT1-K679T mutant on macrophage M1 polarization. STAT1-KO RAW264.7 cells were transfected with the indicated plasmids and then stimulated with 100 ng/ml LPS for 24 h. **A** Immunoblotting analysis of STAT1 Kbhb levels. **B**, **C** RT-qPCR analysis of IL-6 and IL-12 mRNA expression levels. **D**, **E** ELISA was performed to measure the secretion of IL-6 and IL-12. **F** RT-qPCR analysis of iNOS mRNA expression levels. **G**, **H** FCM analysis of the proportion of M1 phenotype (iNOS^+^/F4/80^+^). Data are presented as the mean ± standard deviation of three independent experiments. *p < 0.05 vs Flag-STAT1-WT group and ^#^*p* < 0.05 vs Flag-STAT1-WT + LPS group.

The STAT1-KO RAW264.7 cells were transfected with Flag-STAT1-WT or STAT1-K679T mimetic mutant (lysine residues mutated to threonine, K to T) plasmids, and the results showed that compared to transfection with STAT1-WT plasmids, transfection with STAT1-K679T plasmids increased the STAT1 Kbhb levels in the STAT1-KO RAW264.7 cells in the absence of β-OHB treatment (Fig. 8A). Notably, compared to transfection with the STAT1-WT vector, transfection with the STAT1-K679T plasmids reduced IL-6 and IL-12 mRNA expression and secretion, downregulated iNOS mRNA expression, and decreased the number of M1 macrophages in the LPS-primed STAT1-KO RAW264.7 cells without β-OHB treatment (Fig. 8B–H), suggesting that Kbhb modification of the STAT1 K679 site inhibited M1 macrophage polarization.

### Kbhb modification of the STAT1 K679 site reduced LPS-induced STAT1 phosphorylation and transcriptional activity

**Fig. 9:**
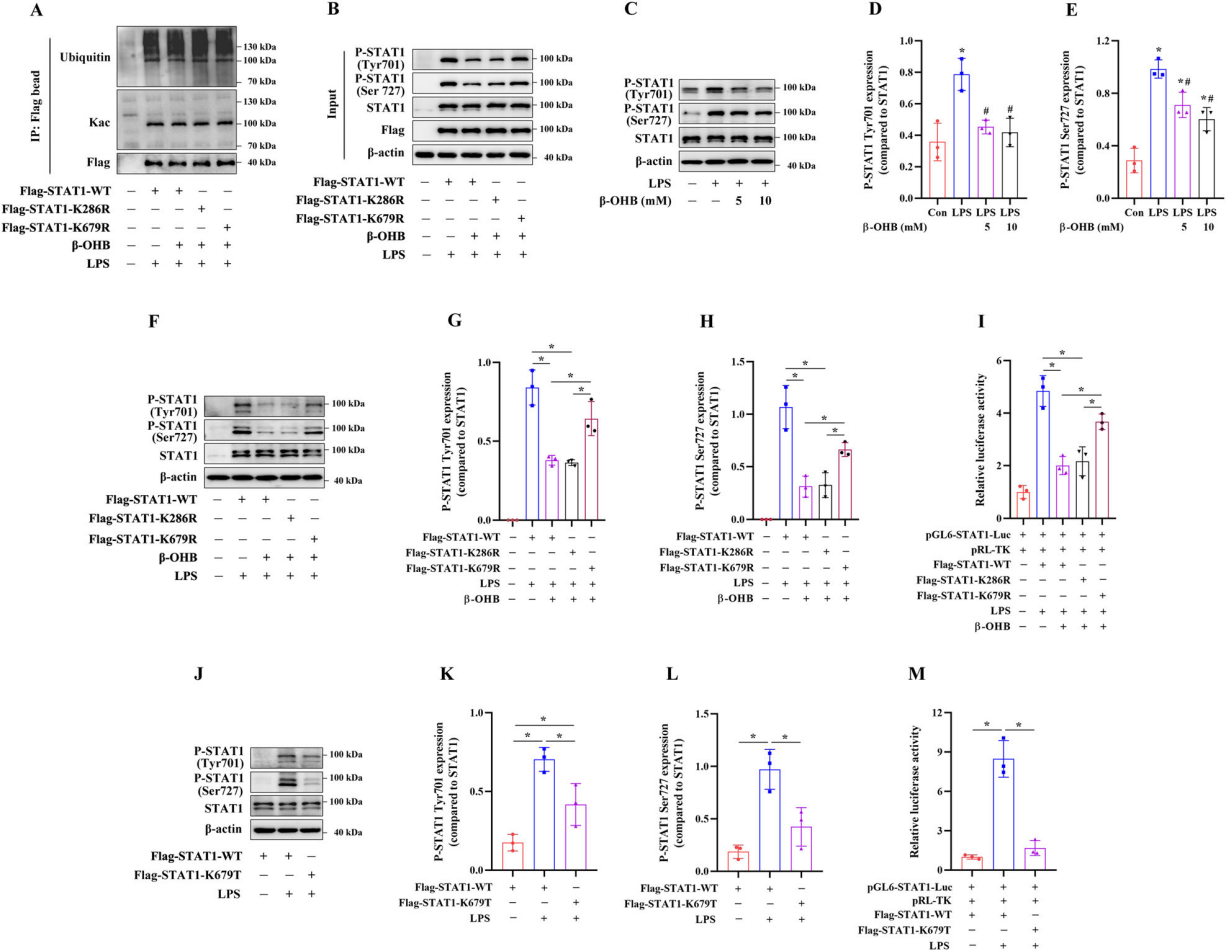
Effects of STAT1 K679 Kbhb modification on the LPS-induced STAT1 phosphorylation and transcriptional activity. HEK293T cells were transfected with the indicated plasmids, treated with 10 mM β-OHB for 24 h, and then stimulated with 100 ng/ml LPS for 4 h. The STAT1-K286R mutant plasmid was used as a negative control. **A** Immunoblotting analysis of the ubiquitination and acetylation levels of STAT1-Flag in the HEK293T cells. **B** Immunoblotting analysis of P-STAT1 expression in the HEK293T cells. RAW264.7 cells were treated with 5 or 10 mM β-OHB for 24 h and then stimulated with 100 ng/ml LPS for 4 h. **C**–**E** Immunoblotting analysis of P-STAT1 expression in the RAW264.7 cells. **F**–**I** STAT1-KO RAW264.7 and HEK293T cells were transfected with the indicated plasmids, treated with 10 mM β-OHB for 24 h, and then stimulated with 100 ng/ml LPS for 4 h. **F**–**H**, **J**–**L** Immunoblotting analysis of P-STAT1 expression in the STAT1-KO RAW264.7 cells. **I**, **M** Dual-luciferase reporter assay of STAT1 transcriptional activity in the HEK293T cells. Data are presented as the mean ± standard deviation of three independent experiments. *p < 0.05 vs Con group and ^#^*p* < 0.05 vs LPS group.

Subsequently, we explored the potential mechanisms by which the Kbhb modification of STAT1 protein regulates M1 macrophage polarization. Kbhb has been demonstrated to alter the phosphorylation or acetylation levels of the protein [18, 41]. Our results revealed that K679R mutation did not affect the ubiquitination and acetylation levels of STAT1 (Fig. 9A), but increased its phosphorylation level in the β-OHB-treated LPS-primed HEK293T cells (Fig. 9B). Treatment with 5 and 10 mM β-OHB significantly decreased the LPS-induced P-STAT1 protein expression in RAW264.7 cells (Fig. 9C–E). Additionally, compared to transfection with the STAT1-WT or STAT1-K286R plasmids, transfection with the STAT1-K679R plasmids upregulated P-STAT1 protein expression in the β-OHB-treated LPS-primed STAT1-KO RAW264.7 cells (Fig. 9F–H) and enhanced STAT1 luciferase activity in the β-OHB-treated LPS-primed HEK293T cells (Fig. 9I). Furthermore, compared to transfection with the STAT1-WT plasmids, transfection with the STAT1-K679T plasmids downregulated P-STAT1 protein expression in the LPS-primed STAT1-KO RAW264.7 cells (Fig. 9J–L) and decreased STAT1 luciferase activity in the LPS-primed HEK293T cells (Fig. 9K). Altogether, these results suggested that Kbhb modification of the STAT1 K679 site played a critical role in the phosphorylation and transcriptional activity of STAT1.

### In vivo effects of Kbhb modification of the STAT1 K679 site

**Fig. 10:**
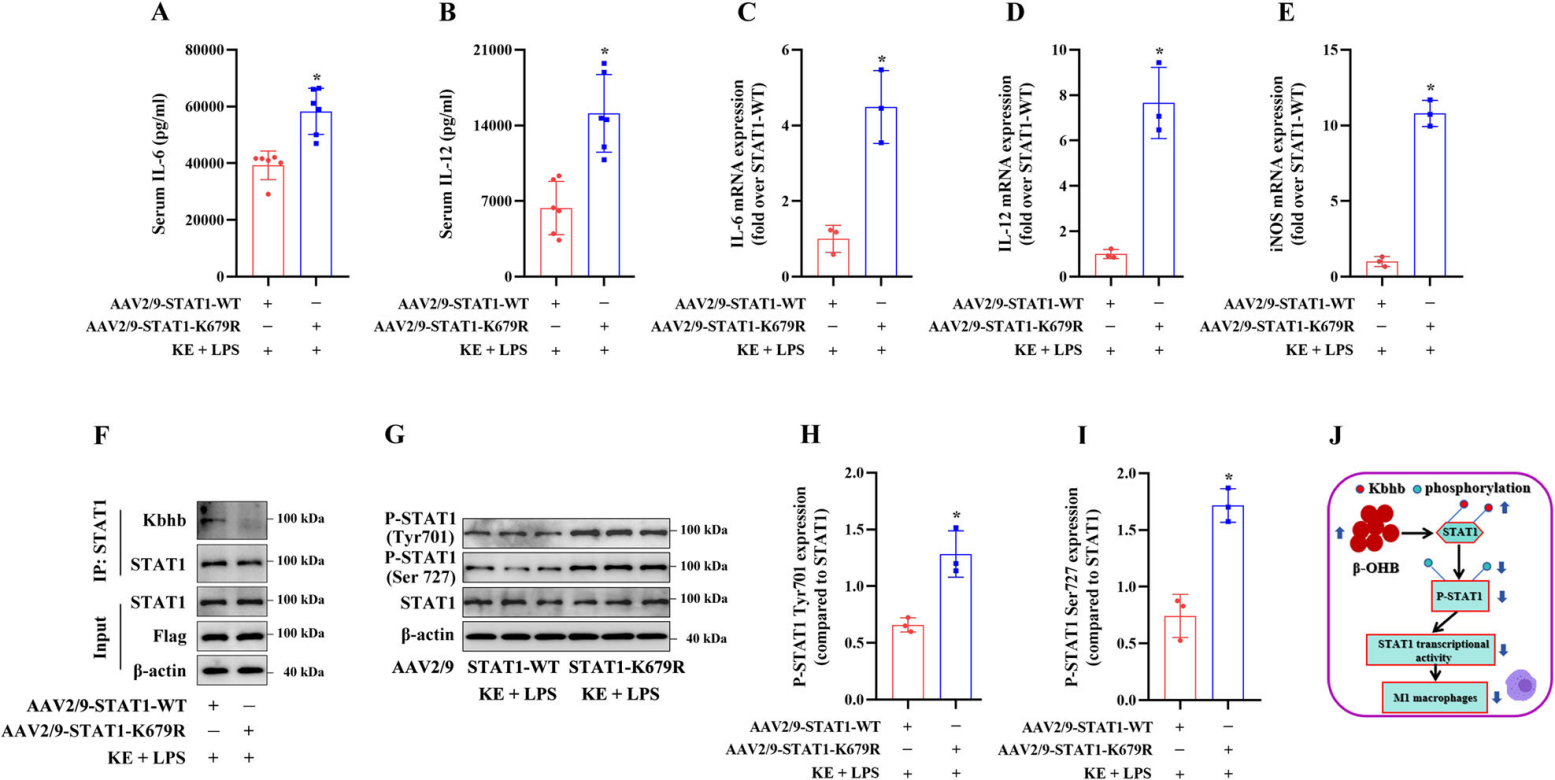
Effects of STAT1 K679 Kbhb modification in vivo. The 9-week-old male C57BL/6 mice were transfected with AAV2/9-STAT1-WT or AAV2/9-STAT1-K679R via intra-bone marrow injection. After 2 weeks of transfection, the mice were subjected to KE + LPS treatment. **A**, **B** ELISA of serum IL-6 and IL-12 levels in mouse PMs (n = 6/group). **C**–**E** RT-qPCR analysis of IL-6, IL-12, and iNOS mRNA expression levels in mouse PMs (n = 3/group). **F**–**I** Immunoblotting analysis of STAT1 Kbhb levels and P-STAT1 expression levels in mouse PMs (n = 3/group). **J** A regulatory model of the β-OHB-induced inhibition of M1 macrophage polarization mediated via STAT1 Kbhb modification. *p < 0.05 vs AAV2/9-STAT1-WT group.

To investigate the effects of Kbhb modification of the STAT1 K679 site in vivo, we transfected C57BL/6 J mice with AAV2/9-m-STAT1-Flag or AAV2/9-m-STAT1-K679R-Flag vector via intra-bone marrow injection. The results revealed that compared to transfection with the STAT1-WT vector, transfection with the K679R mutant vector significantly increased the serum levels of IL-6 and IL-12 in KE + LPS-treated mice (Fig. 10A, B), as well as the expression of IL-6, IL-12, and iNOS mRNA in PMs isolated from these mice (Fig. 10C–E). Additionally, compared to transfection with the STAT1-WT vector, transfection with the K679R mutant vector decreased STAT1 Kbhb levels and significantly increased P-STAT1 protein expression in the PMs isolated from the KE + LPS-treated mice (Fig. 10F–I).

## Discussion

Ketone bodies are primarily synthesized in the mitochondria of liver cells, using acetyl-CoA produced from the beta-oxidation of fatty acids. β-OHB, AcAc, and acetone constitute 78%, 20%, and 2% of the ketone bodies in the body, respectively. β-OHB is more stable than AcAc and serves as an alternative energy source and a crucial signaling molecule [5]. Previous studies found that β-OHB reduces pro-inflammatory cytokine production in macrophages [12–14]. Consistently, in the present study, we found that β-OHB treatment reduced IL-6 and IL-12 production in the LPS-primed BMDMs and RAW264.7 cells. For in vivo analysis, we administered the mice directly with exogenous KE instead of subjecting them to a ketogenic diet, to investigate the effect of elevated β-OHB levels on the inflammatory factor production in mouse PMs, without altering the metabolic pathway [42]. Consistent with the results of in vitro experiments, KE treatment significantly downregulated the LPS-induced IL-6 and IL-12 expression in mouse PMs. Additionally, quantitative proteomic analysis revealed that β-OHB treatment decreased the expression of M1 polarization-related proteins in BMDMs. In addition, β-OHB treatment significantly inhibited LPS-induced M1 polarization of BMDMs, RAW264.7 cells, and mouse PMs. These results demonstrate an association between β-OHB and the inhibition of M1 macrophage polarization.

In this study, we aimed to investigate the underlying molecular mechanisms of the β-OHB-mediated inhibitory effects on M1 macrophage polarization. β-OHB induces Kbhb modification of proteins, which plays a crucial role in various physiological and pathological processes [16–18]. Our results revealed that β-OHB treatment induced Kbhb modification in BMDMs, RAW264.7 cells, PMA differentiated-THP-1 macrophages, and KCs, while both 48-h starvation and KE administration induced Kbhb modification in mouse PMs. Moreover, our results showed that AcAc treatment did not induce Kbhb modification and that β-OHB treatment did not induce Kac and Kla modifications. These results indicate that β-OHB specifically induces Kbhb modifications in macrophages, both in vitro and in vivo.

In this study, we induced Kbhb modification in RAW264.7 cells and mouse BMCs by β-OHB incubation and KE administration, respectively, and assessed its persistence and related functions under β-OHB-free culture/differentiation conditions. The results revealed that even after 48-h under β-OHB-free culture conditions, the Kbhb modification persisted in RAW264.7 cells and continued to reduce its IL-6, IL-12, and iNOS mRNA expression. Similarly, Kbhb modification persisted in the KE-treated mouse BMDMs and inhibited its IL-6, IL-12, and iNOS mRNA expression. Altogether, these results demonstrated that Kbhb modification persisted even after the withdrawal of β-OHB and continued to exert its anti-inflammatory effects. This provides indirect evidence for the regulatory role of Kbhb modifications in macrophage M1 polarization.

BDH1 plays a pivotal role in the metabolism of ketone bodies [5]; however, the lack of BDH1 in macrophages may lead to the accumulation of β-OHB within the cells, making it prone to Kbhb modification in macrophages. Overexpression of BDH1 did not affect the inflammatory phenotype of BMDMs. However, BDH1 overexpression led to a reduction in β-OHB levels in β-OHB-treated BMDMs, decreased Kbhb modification, and influenced the polarization phenotype. This provides direct evidence that Kbhb modification regulates macrophage M1 polarization.

Global Kbhb proteomic analysis identified 1549 proteins in the β-OHB-treated BMDMs that showed significant upregulation of Kbhb modification. GO enrichment analysis of these Kbhb-modified proteins showed that they were significantly enriched in IL-12 response and Fc-gamma receptor signaling pathway, which are essential for M1 macrophage programming [34, 36]. Among the Kbhb-modified proteins, the level of Kbhb modification on the STAT1 transcription factor was notably increased in the β-OHB-treated BMDMs. MS analysis revealed the presence of six Kbhb sites on STAT1, among which the K679 site, was identified as the primary Kbhb modification site through site-directed mutation experiments.

STAT1 is a key signaling molecule that determines the M1 phenotype of macrophages. Upon activation of the STAT1 signaling pathway, P-STAT1 translocates from the cytoplasm to the nucleus, promoting the transcription of its target genes, including IL-12 and iNOS [37, 43]. In this study, β-OHB treatment significantly decreased the LPS-induced P-STAT1 protein expression in RAW264.7 cells. Site-directed mutation analysis of STAT1 K679 revealed that β-OHB-mediated STAT1 Kbhb at the K679 site reduced LPS-induced STAT1 phosphorylation and transcriptional activity.

This study has several limitations. First, only one of the Kbhb-modified DEPs was investigated in this study, thus warranting further investigation of the other DEPs which may play a crucial role in regulating M1 macrophage polarization. Second, the β-OHB-induced inhibition of STAT1 phosphorylation and transcriptional activity may be mediated by disrupting STAT1 dimer formation [44] and/or by inhibiting STAT1–ligand binding [45, 46], which needs to be further investigated. Third, although we have confirmed that β-OHB inhibited LPS-induced mouse sepsis and the STAT1-K679R mutation weakened the anti-inflammatory effects of β-OHB in vivo (Fig. 10A–E), further research focus on constructing conditional myeloid cell-specific point mutation mouse (STAT1 p.K679R Lyz2-cre) to elucidate the significance of Kbhb modifications in the treatment of relevant inflammatory diseases, such as septic liver injury or obesity, is needed.

In conclusion, this is the first study to demonstrate the role of β-OHB in inhibiting M1 macrophage polarization and inducing macrophage Kbhb modification. Our study showed that β-OHB mediates its inhibitory effects by inducing Kbhb modification of the STAT1 K679 site, thereby inhibiting its LPS-induced phosphorylation and transcriptional activity (Fig. 10J).

## Supplementary information

Supplementary data

WB original image

## Data Availability

The datasets generated during the current study are available from the corresponding author on reasonable request.
