# Supplementary material for: β-Hydroxybutyrate suppresses M1 macrophage polarization through β-hydroxybutyrylation of the STAT1 protein
Source: Cell Death Dis. 2024 Dec 3;15(12):874. doi: 10.1038/s41419-024-07268-3 (PMC11615246; doi:10.1038/s41419-024-07268-3)
Supplement: Supplementary file 1 — Supplementary data [file 41419_2024_7268_MOESM1_ESM.docx]

**SUPPLEMENTARY DATA**

**Proteomics analysis**

**1.1 Protein Extraction**

The sample was grinded with liquid nitrogen into cell powder and then transferred to a 5-ml centrifuge tube. After that, four volumes of pre-cooled 10% TCA/acetone was added to the cell powder, precipitated at -20℃ for 4 h. The precipitate was washed 3 times with the pre-cooled acetone. After that, four volumes of lysis buffer (8 M urea, 1% protease inhibitor cocktail) was added to the protein precipitate, followed by sonication 3 min on ice using a high intensity ultrasonic processor (Scientz). The remaining debris was removed by centrifugation at 12,000 g at 4 °C for 10 min. Finally, the supernatant was collected and theprotein concentration was determined with BCA kit according to the manufacturer’s instructions.

**1.2 Trypsin Digestion**

The sample was slowly added to the final concentration of 20% (m/v) TCA to precipitate protein, then vortexed to mix and incubated for 2 h at 4 °C. The precipitate was collected by centrifugation at 4500 g for 5 min at 4 °C. The precipitated protein was washed with pre-cooled acetone for 3 times and dried for 1 min. The protein sample was then redissolved in 200 mM TEAB and ultrasonically dispersed. Trypsin was added at 1:50 trypsin-to-protein mass ratio for the first digestion overnight. The sample was reduced with 5 mM dithiothreitol for 30 min at 56 °C and alkylated with 11 mM iodoacetamide for 15 min at room temperature in darkness. Finally, the peptides were desalted by Strata X SPE column.

**1.3 Mass Spectrometer**

The tryptic peptides were dissolved in solvent A, directly loaded onto a home-made reversed-phase analytical column (25-cm length, 100 μm i.d.). The mobile phase consisted of solvent A (0.1% formic acid, 2% acetonitrile/in water) and solvent B (0.1% formic acid in acetonitrile). Peptides were separated with following gradient: 0-70 min, 6%-24%B; 70-82 min, 24%-35%B; 82-86 min, 35%-80%B; 86-90 min, 80%B, and all at a constant flow rate of 450 nl/min on a NanoElute UHPLC system (Bruker Daltonics). The peptides were subjected to capillary source followed by the timsTOF Pro mass spectrometry. The electrospray voltage applied was 1.60 kV. Precursors and fragments were analyzed at the TOF detector, with a MS/MS scan range from 100-1700. The timsTOF Pro was operated in parallel accumulation serial fragmentation (PASEF) mode. Precursors with charge states 0-5 were selected for fragmentation, and 10PASEF-MS/MS scans were acquired per cycle. The dynamic exclusion was set to 30 s.

**1.4 Database Search**

The resulting MS/MS data were processed using MaxQuant search engine (v.1.6.15.0). Tandem mass spectra were searched against Mus_musculus_10090_SP_ 20230103.fasta (17132 entries) concatenated with reverse decoy and contaminants database. Trypsin/P was specified as cleavage enzyme allowing up to 2 missing cleavages. Min. peptide length was set as 7 and max. number of modification per peptide was set as 5. The mass tolerance for precursor ions was set as 20 ppm in first search and 20 ppm in main search, and the mass tolerance for fragment ions was set as 20 ppm. Carbamidomethyl on Cys was specified as fixed modification, and acetylation on protein N-terminal and oxidation on Met were specified as variable modifications. False discovery rate (FDR) of protein, peptide and PSM was adjusted to < 1%.


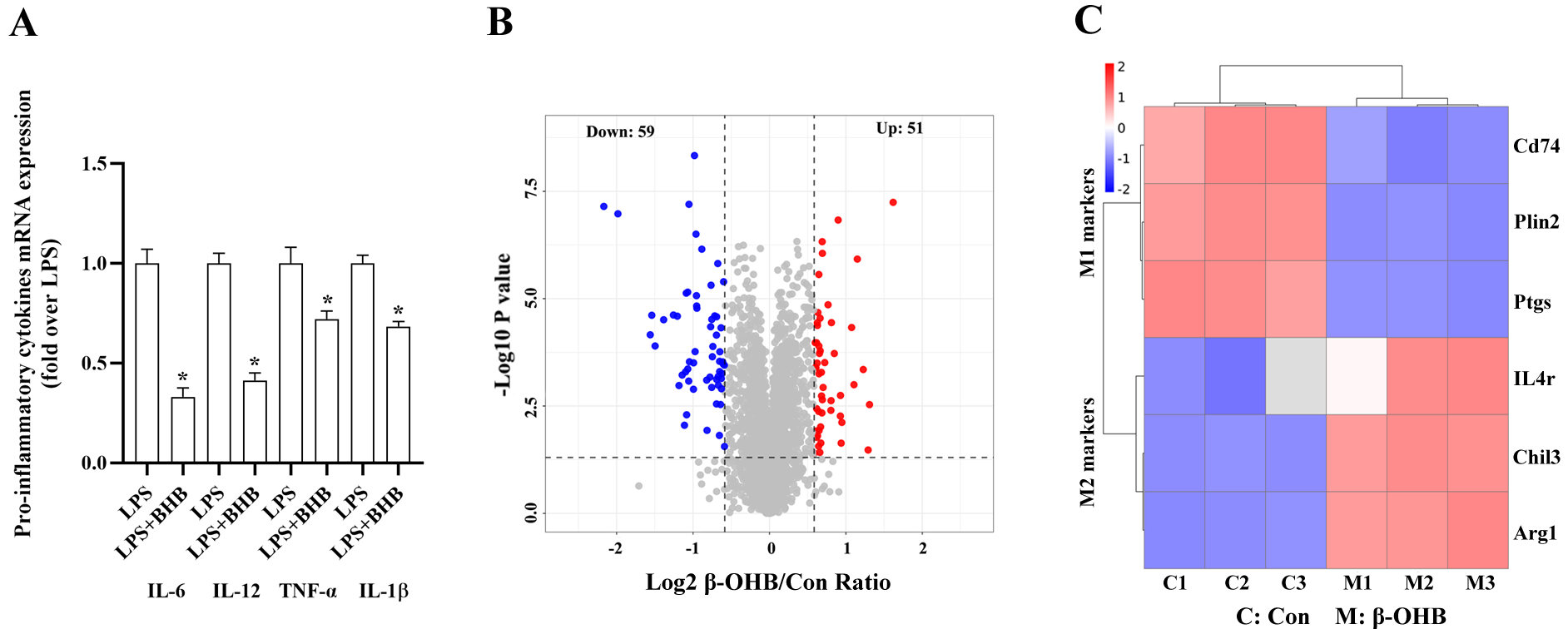


**Fig. S1 Effects of β-OHB treatment on pro-inflammatory cytokines expression and differential protein expression analysis in BMDMs.** (**A)** BMDMs were treated with 10 mM β-OHB for 24 h and then stimulated with 100 ng/ml LPS for 24 h. RT-qPCR analysis of IL-6, IL-12, TNF-α, and IL-1β mRNA expression levels in the BMDMs (n = 3/group). Data are presented as the mean ± standard deviation. **p* < 0.05 vs LPS group. BMDMs were incubated in the presence or absence of 10 mM β-OHB for 24 h and then subjected to proteomics analysis. The experiments were conducted in triplicates. (**B)** The volcano plots of DEPs in the β-OHB-incubated and control BMDMs. (**C)** Heatmaps of the selected DEPs.


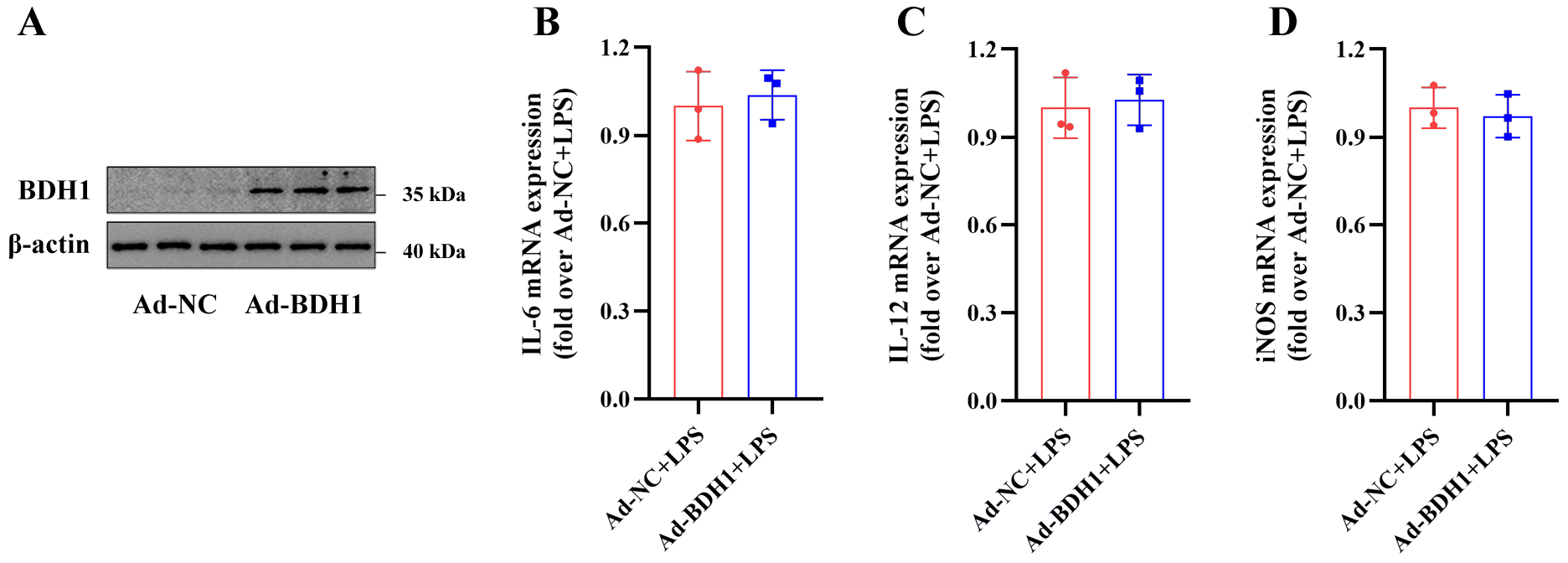


**Fig. S2 Effects of BDH1 overexpression on IL-6, IL-12, and iNOS expression in BMDMs.** BMDMs were infected with Ad-normal control (NC) or Ad-BDH1. **(A)** The efficiency of overexpression for Ad-BDH1 was assessed. Following the infection, BMDMs were stimulated with 100 ng/ml LPS for 24 h. (**B**–**D)** RT-qPCR analysis of IL-6, IL-12, and iNOS mRNA expression levels in the BMDMs (n = 3/group). **p* < 0.05 vs Ad-NC + LPS group.


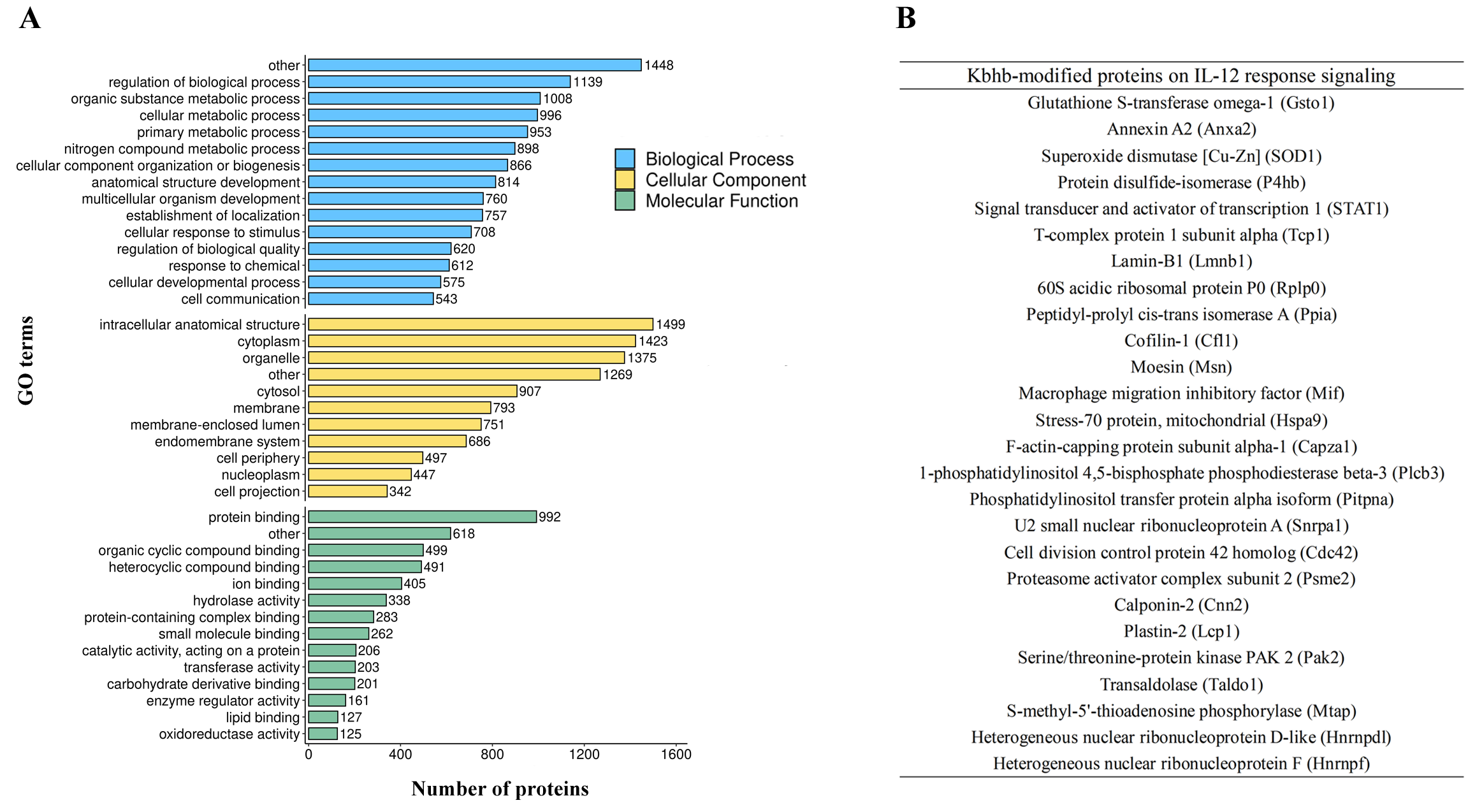


**Fig. S3 GO functional enrichment analysis of differential Kbhb-modified proteins (A) and Kbhb-modified proteins on IL-12 response signaling (B).**

**
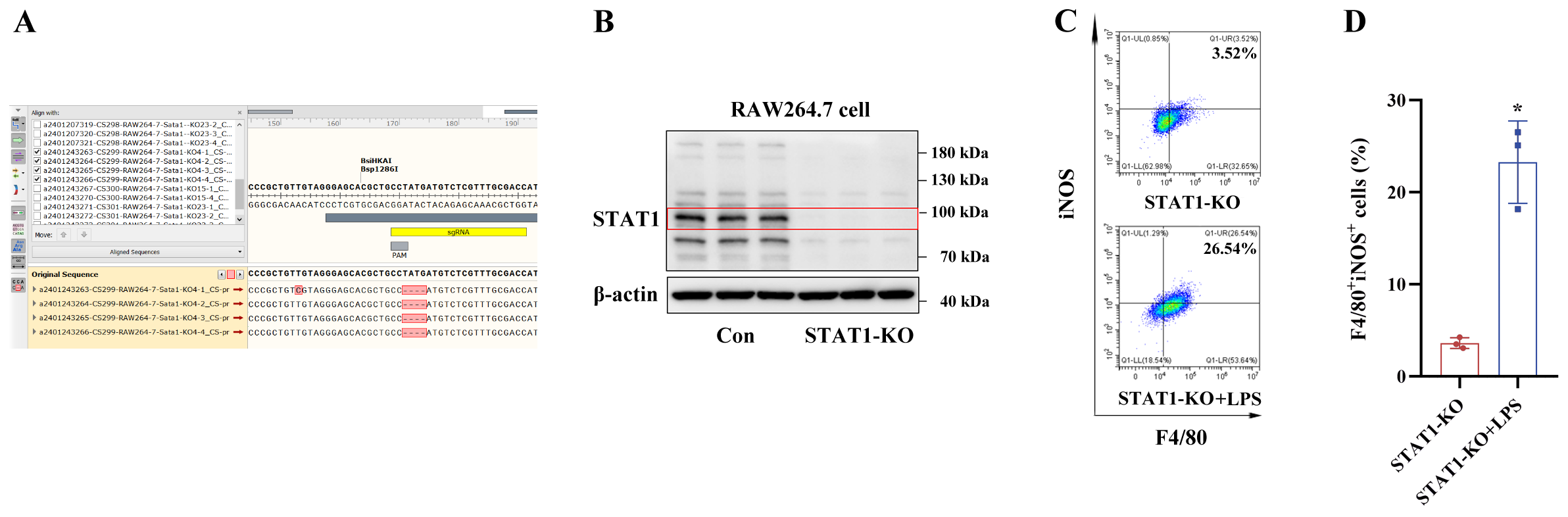
**

**Fig. S4 Construction of STAT1-KO RAW264.7 cells and the effects of STAT1-KO on macrophage M1 polarization. (A)** Sequencing results of the STAT1-KO RAW264.7 cells indicated the presence of a 4-bp deletion in the STAT1 gene. (**B)** Immunoblotting results confirmed the successful KO of STAT1. The STAT1-KO RAW264.7 cells were stimulated with 100 ng/ml LPS for 24 h. (**C**, **D)** FCM analysis of the proportion of M1 phenotype (iNOS^+^/F4/80^+^) STAT1-KO RAW264.7 cells. Data are presented as the mean ± standard deviation of three independent experiments. **p* < 0.05 vs STAT1-KO cells.
