# Supplementary material for: β-Hydroxybutyrate suppresses M1 macrophage polarization through β-hydroxybutyrylation of the STAT1 protein
Source: Cell Death Dis. 2024 Dec 3;15(12):874. doi: 10.1038/s41419-024-07268-3 (PMC11615246; doi:10.1038/s41419-024-07268-3)
Supplement: Supplementary file 2 — WB original image [file 41419_2024_7268_MOESM2_ESM.pdf]

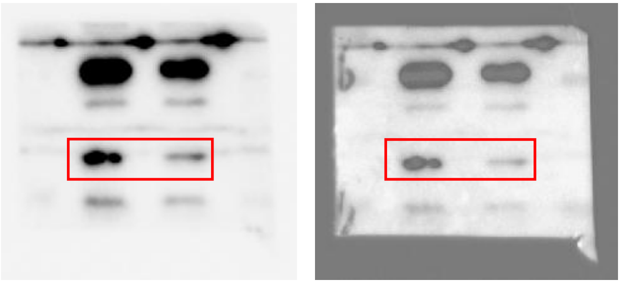

Figure 1. E IL-6

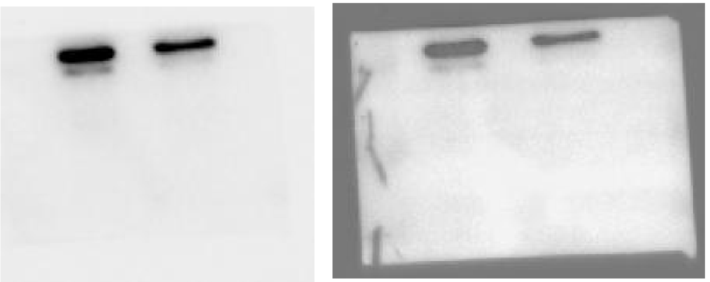

Figure 1. E IL-12

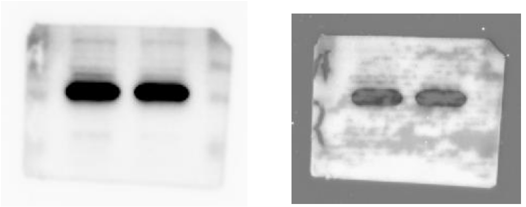

Figure 1. E actin

Figure 1. E IL-6 and IL-12 protein expression in the BMDMs

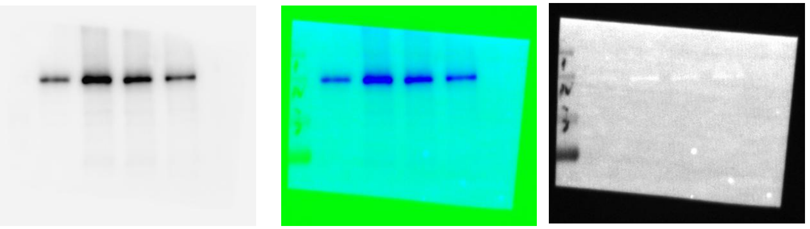

Figure 2. A iNOS

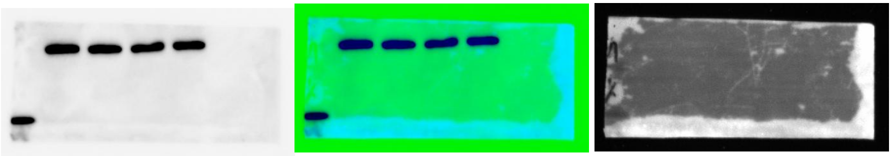

Figure 2. A actin

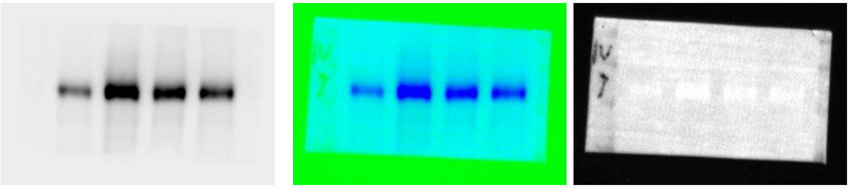

Figure 2. E iNOS

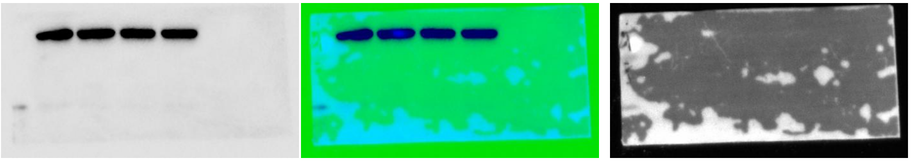

Figure 2. E actin

Figure 2. Immunoblotting analysis of iNOS protein expression in the BMDMs A and RAW264.7 cells E

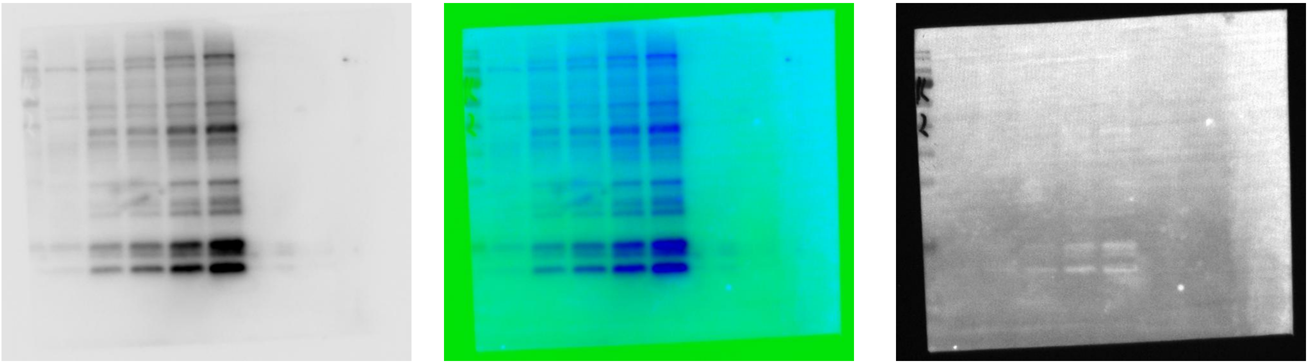

Figure 3. A Kbhb

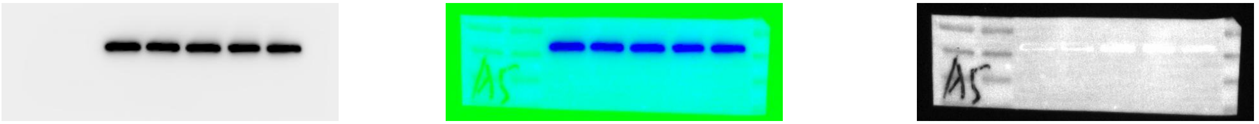

Figure 3. A actin

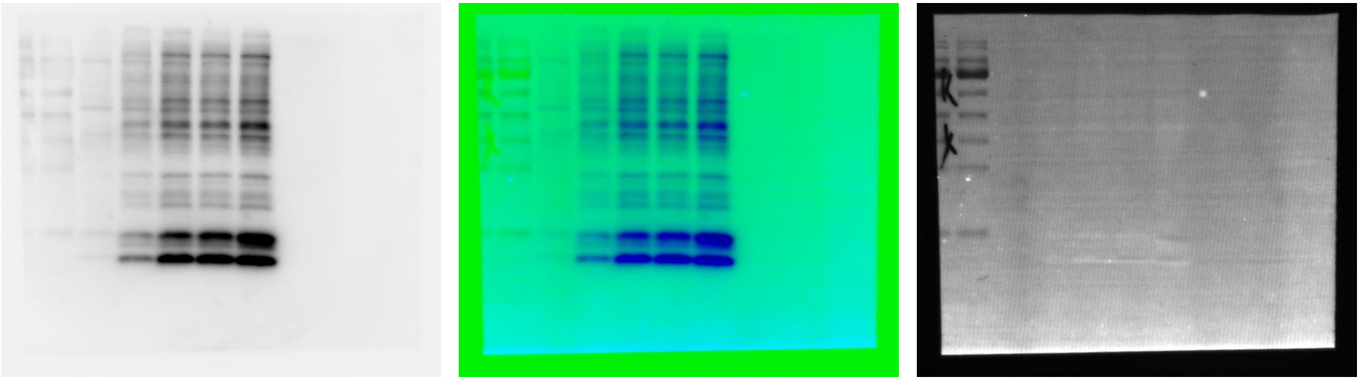

Figure 3. B Kbhb

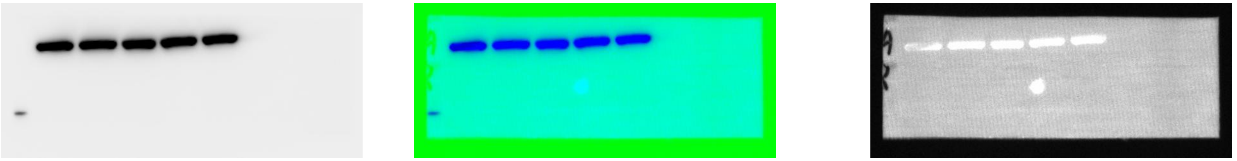

Figure 3. B actin

Figure 3. A RAW264.7 cells were treated with β-OHB at the indicated concentrations for 24 h
B RAW264.7 cells were treated with 10 mM β-OHB at the indicated times

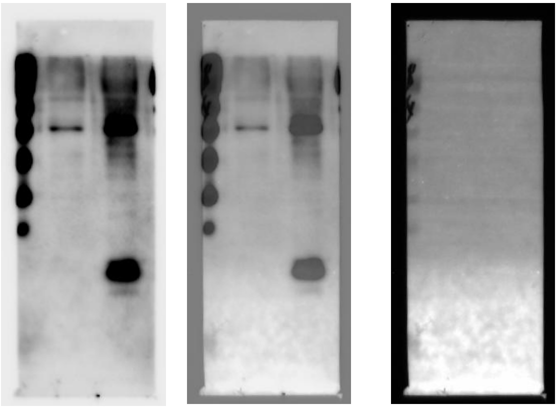

Figure 3. C Kbhb

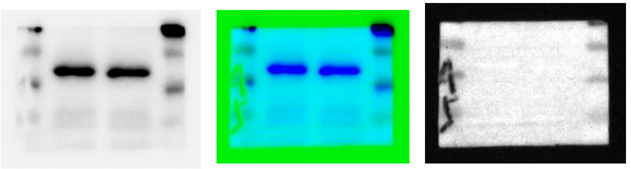

Figure 3. C actin

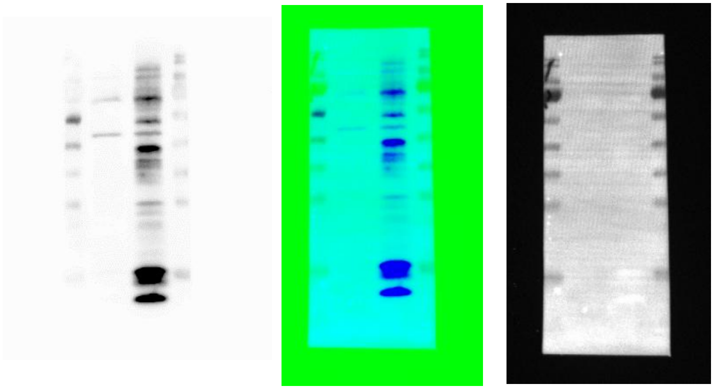

Figure 3. D Kbhb

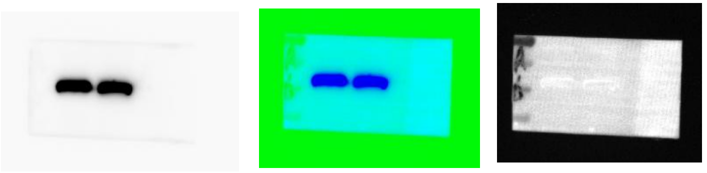

Figure 3. D actin

Figure 3. C BMDMs were treated with 10 mM β-OHB for 24 h

D Immunoblotting analysis of Kbhb modification in the PMA differentiated-THP-1 macrophages

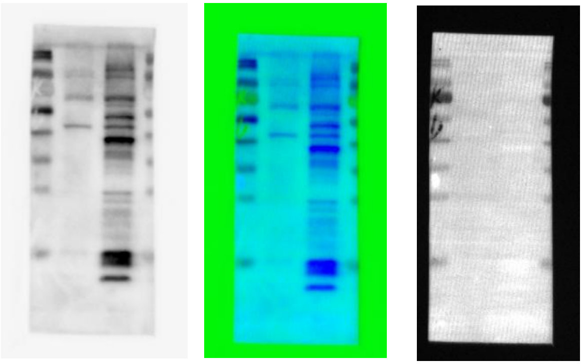

Figure 3. E Kbhb

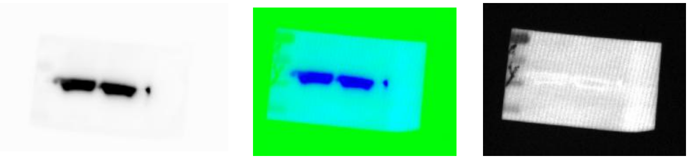

Figure 3. E actin

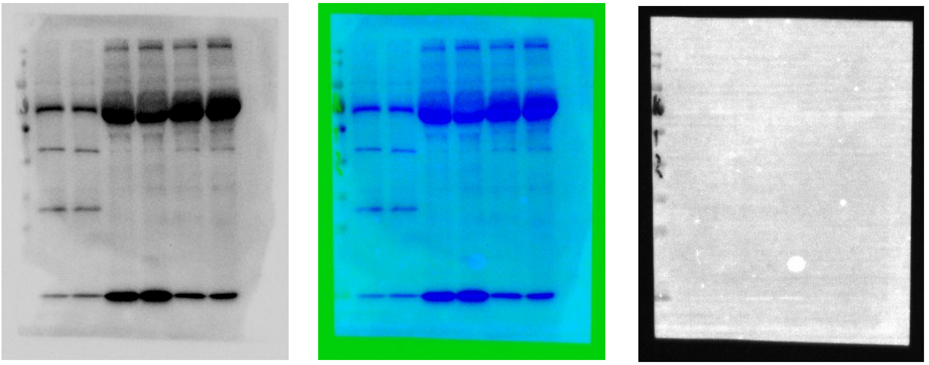

Figure 3. G Kbhb

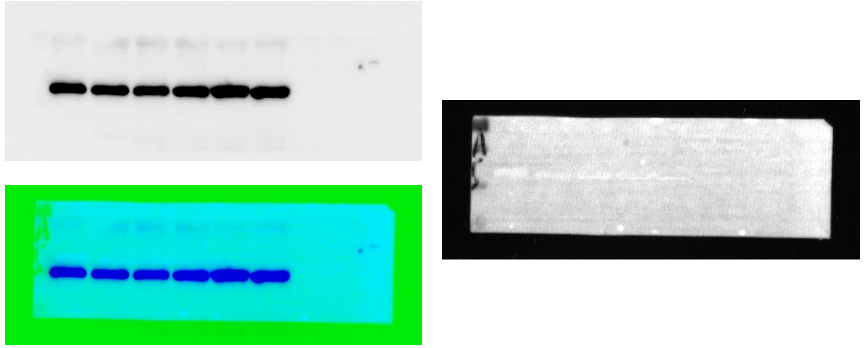

Figure 3. G actin

Figure 3. E Immunoblotting analysis of Kbhb modification in the mouse KCs
G Immunoblotting analysis of Kbhb modification in the mouse PMs

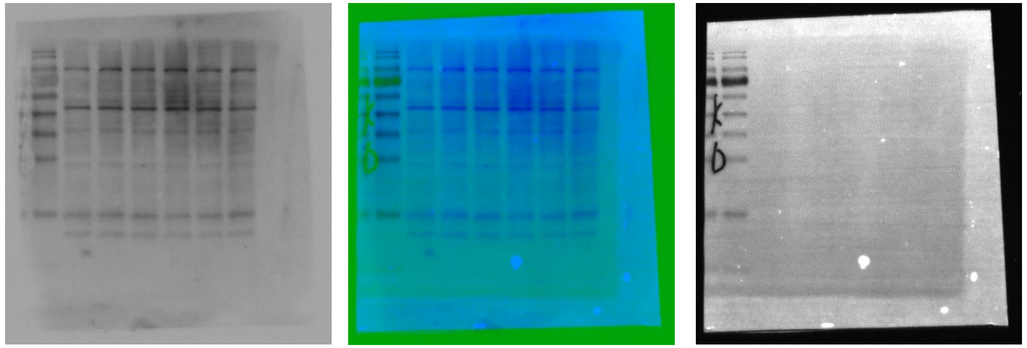

Figure 3. H Kbhb

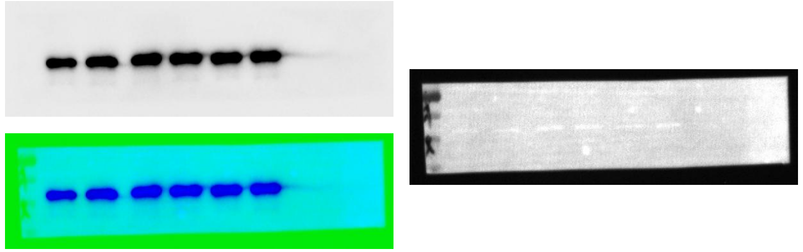

Figure 3. H actin

Figure 3. H Immunoblotting analysis of the Kbhb modification in the AcAc-treated RAW264.7 cells

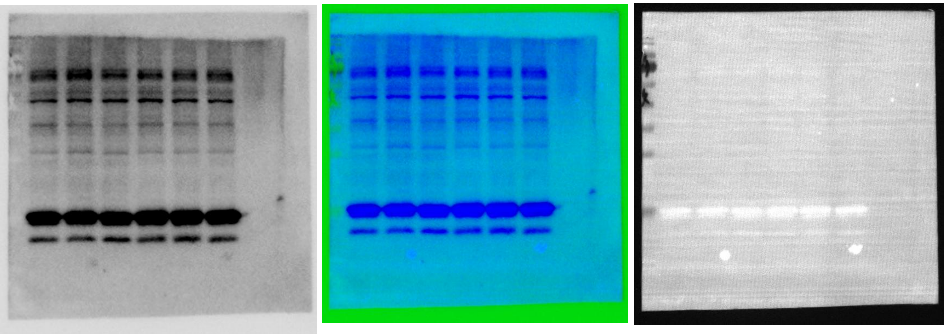

Figure 3. I Kac

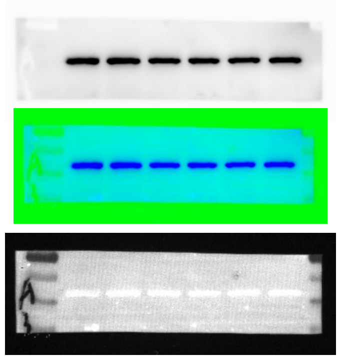

Figure 3. I actin

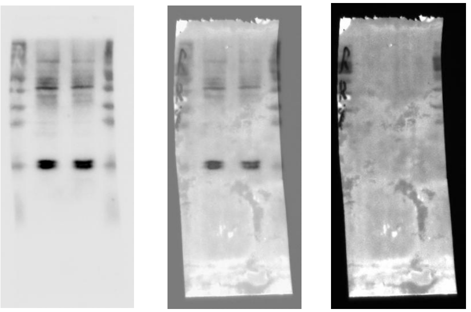

Figure 3. J Kla

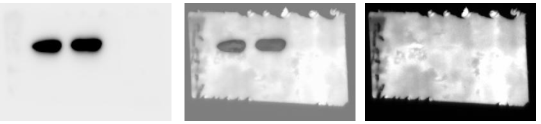

Figure 3. J actin

Figure 3. Immunoblotting analysis of Kac I and Kla J PTMs in β-OHB-treated RAW264.7 cells

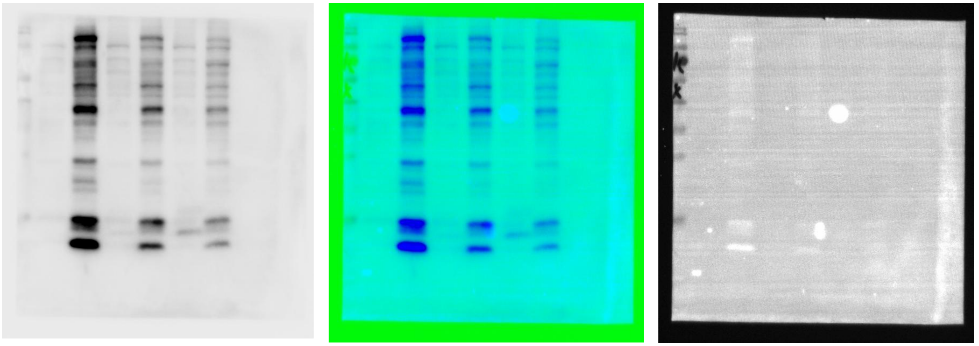

Figure 4. A Kbhb

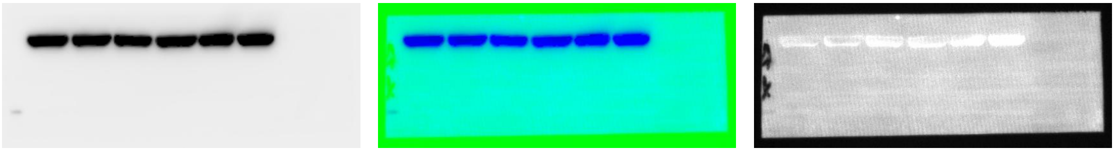

Figure 4. A actin

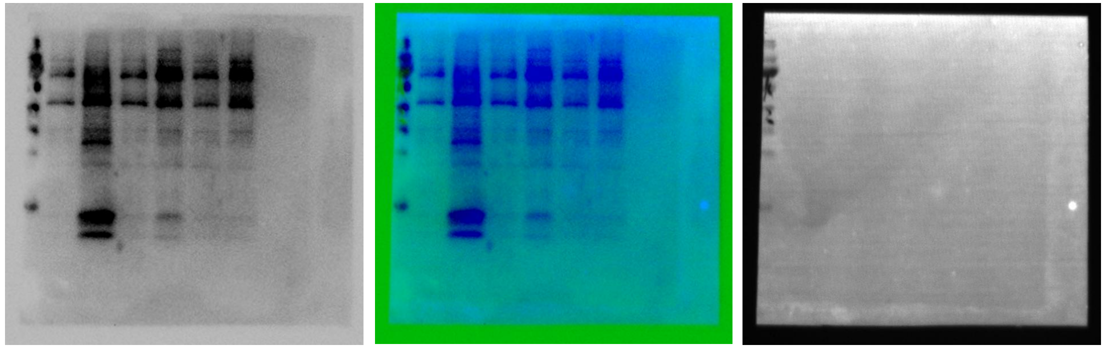

Figure 4. G Kbhb

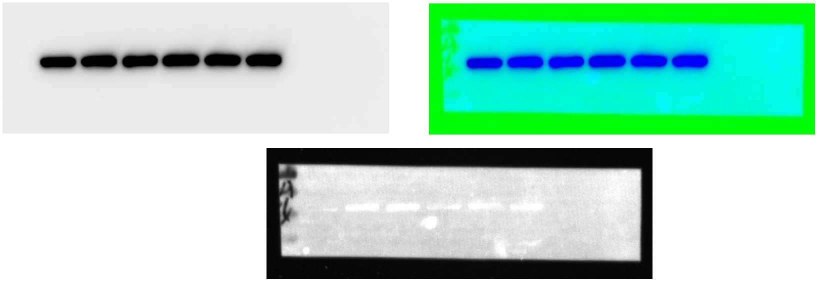

Figure 4. G actin

Figure 4. A Immunoblotting analysis of the Kbhb modification in the RAW264.7 cells
G Immunoblotting analysis of the Kbhb modification in the BMCs treated with LCS at the indicated times

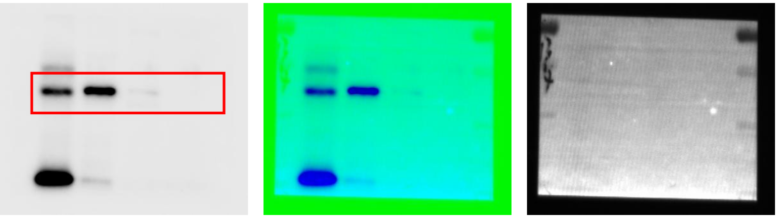

Figure 4. K BDH1

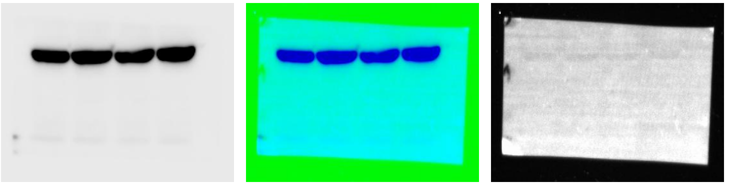

Figure 4. K actin

Figure 4. K BDH1 protein expression in mice liver lysates, HepG2 cells, BMDMs, and PMs.

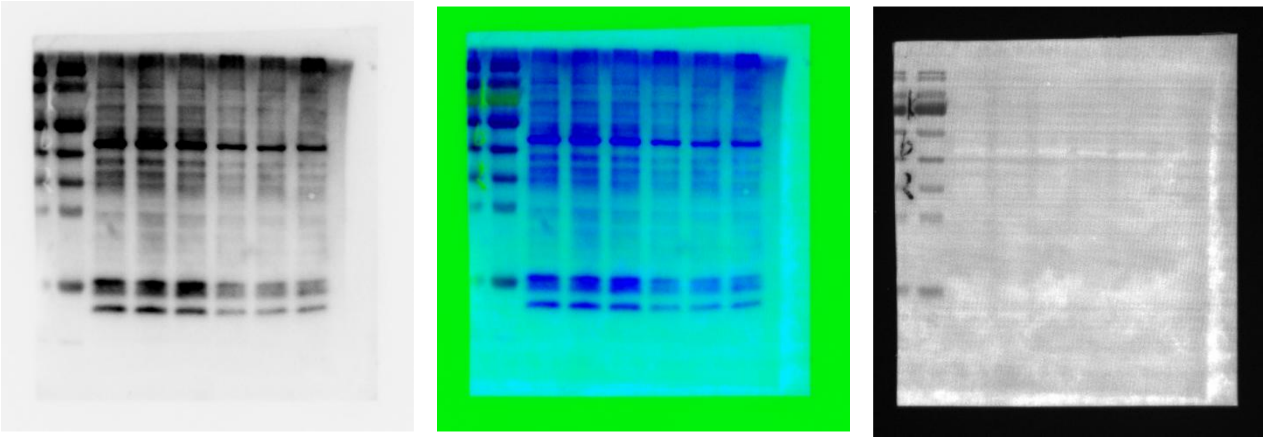

Figure 4. N Kbhb

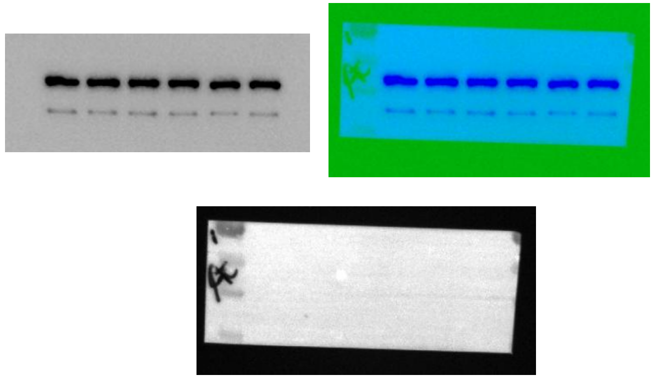

Figure 4. N actin

Figure 4. N Immunoblotting analysis of the Kbhb modification in the BMDMs.

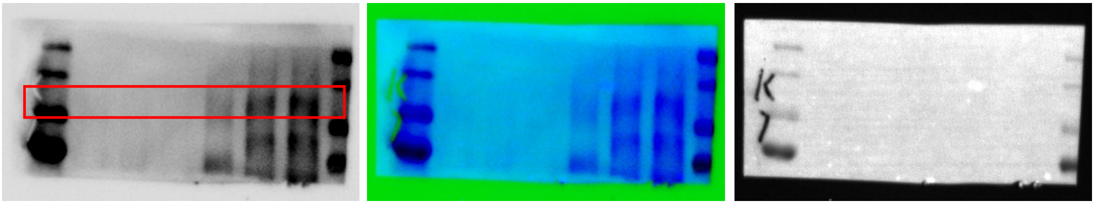

Figure 6. B Kbhb

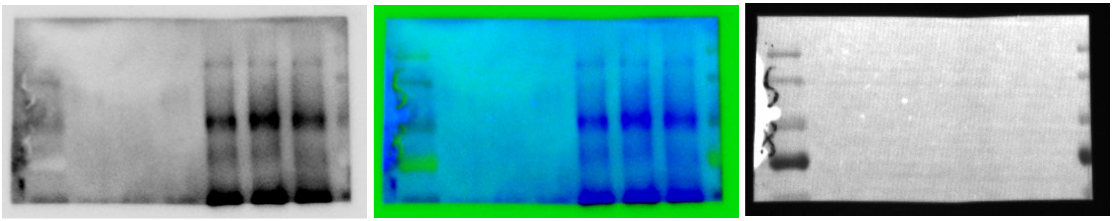

Figure 6. B STAT1

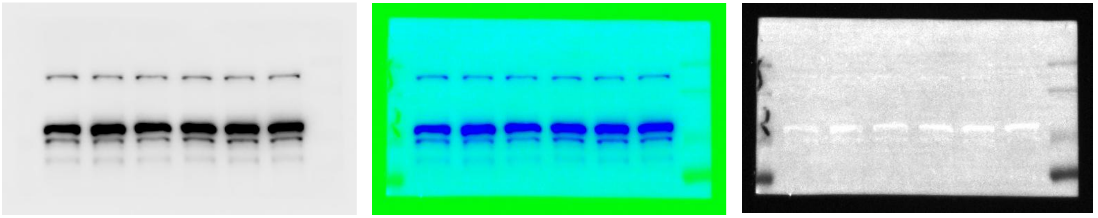

Figure 6. B STAT1

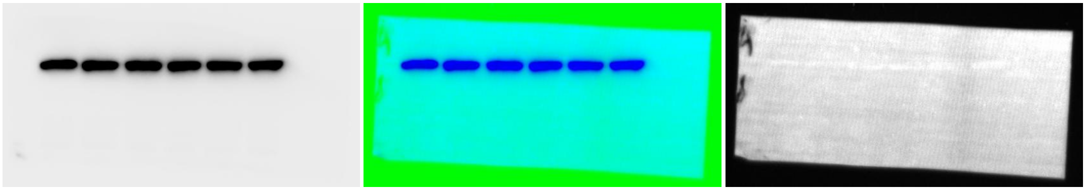

Figure 6. B actin

Figure 6. B RAW264.7 cells were treated with or without 5 or 10 mM β-OHB for 24 h. Immunoblotting analysis of STAT1 Kbhb levels in RAW264.7 cells.

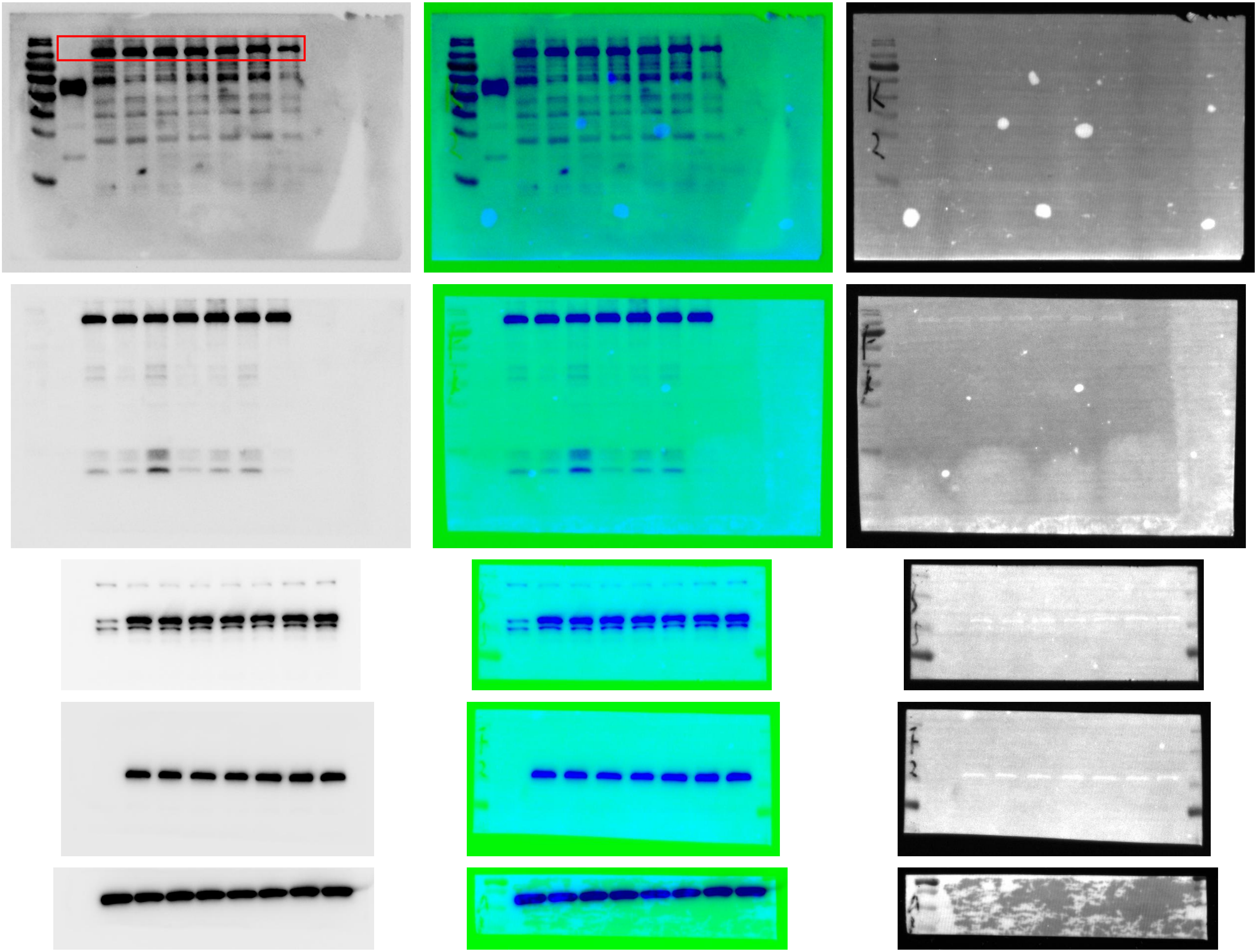

Figure 6. C Kbhb

Figure 6. C Flag

Figure 6. C STAT1

Figure 6. C Flag

Figure 6. C actin

Figure 6. C HEK293T cells were transfected with the indicated plasmids and treated with 10 mM β-OHB for 24 h. Immunoblotting analysis of STAT1 Kbhb levels in HEK293T cells.

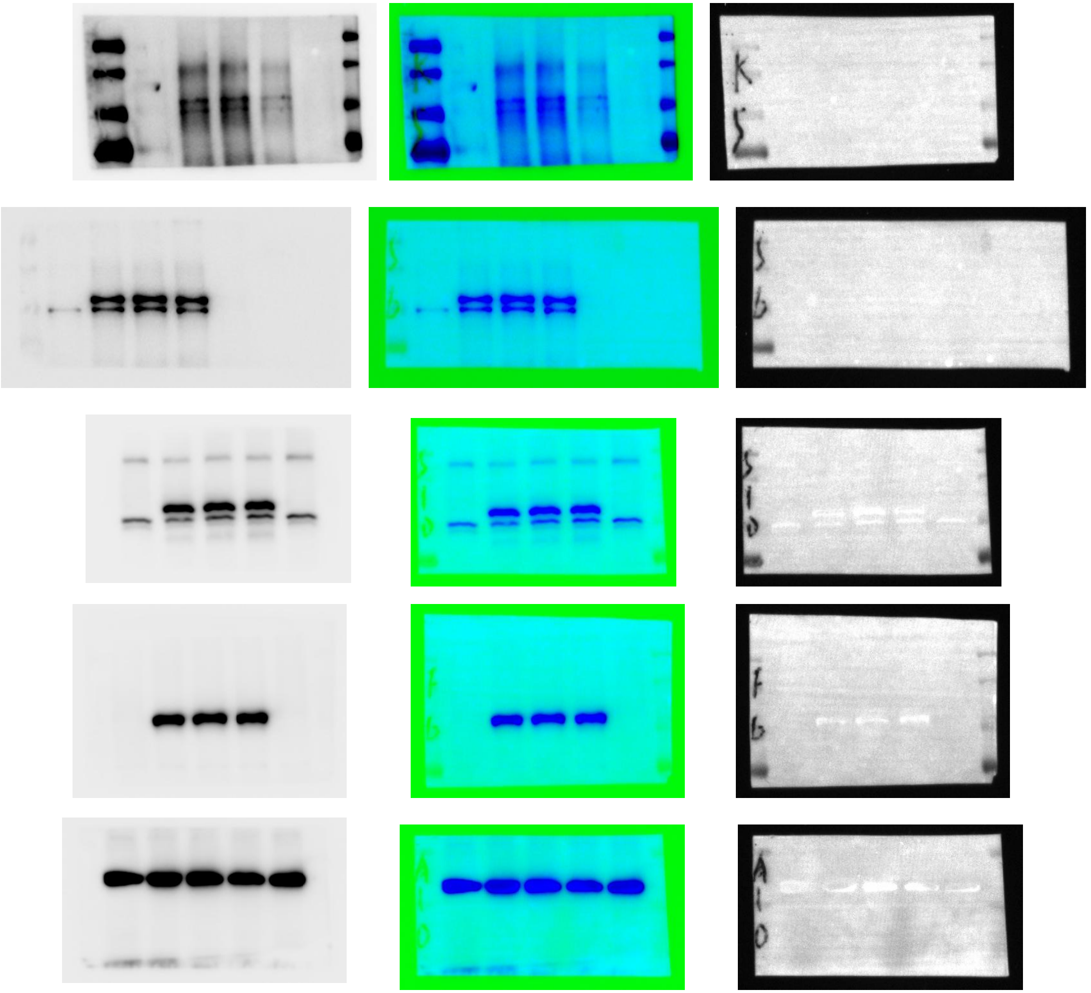

Figure 6. D Kbhh

Figure 6. D STAT1

Figure 6. D STAT1

Figure 6. D Flag

Figure 6. D actin

Figure 6. D HEK293T cells were transfected with the indicated plasmids and treated with 10 mM β-OHB for 24 h. Immunoblotting analysis of STAT1 Kbhh levels in HEK293T cells.

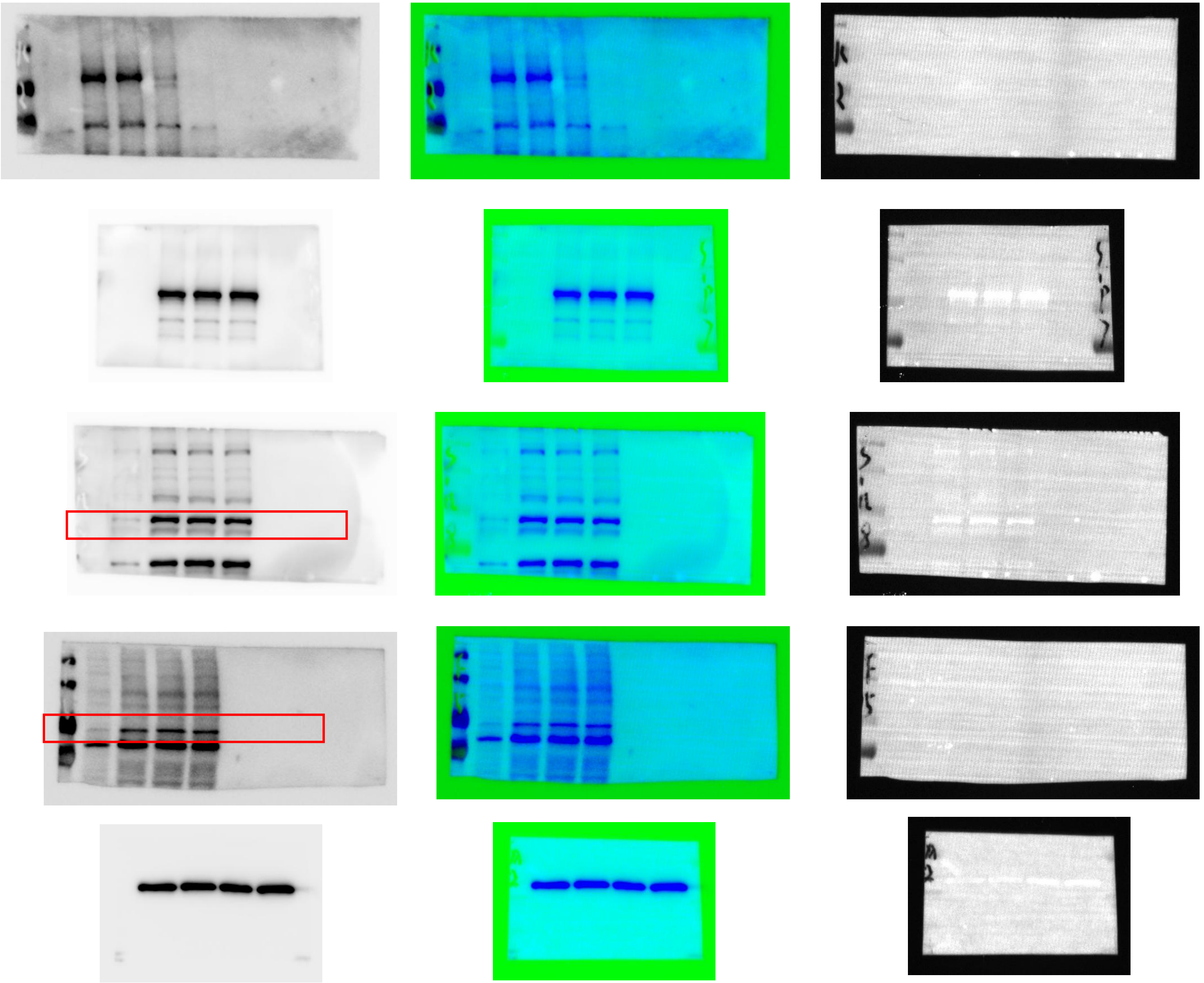

Figure 7. A Kbhb

Figure 7. A STAT1

Figure 7. A STAT1

Figure 7. A Flag

Figure 7. A actin

Figure 7. A Immunoblotting analysis of STAT1 Kbhb levels in the STAT1-KO RAW264.7 cells.

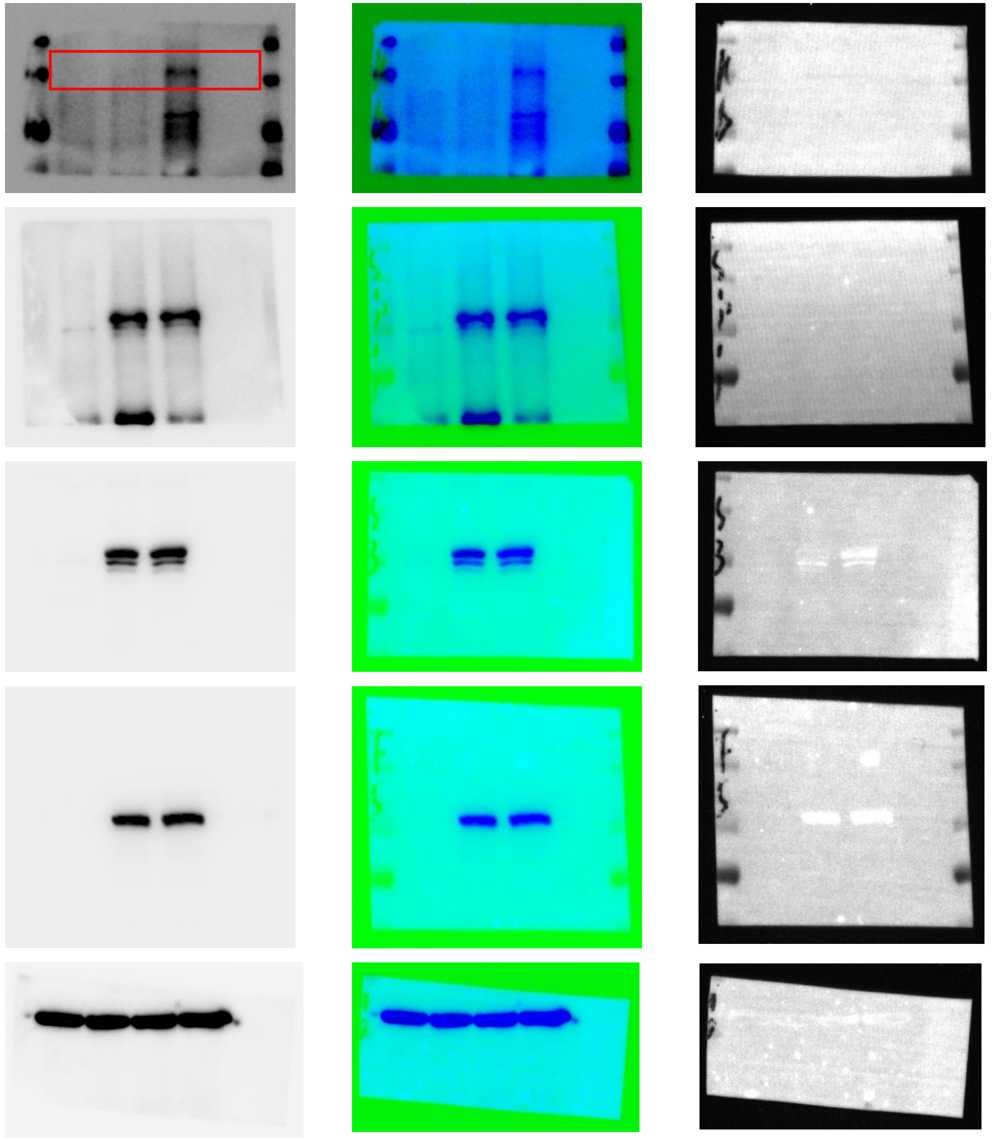

Figure 8. A Kbhh

Figure 8. A STAT1

Figure 8. A STAT1

Figure 8. A Flag

Figure 8. A actin

Figure 8. A Immunoblotting analysis of STAT1 Kbhh levels in the STAT1-KO RAW264.7 cells.

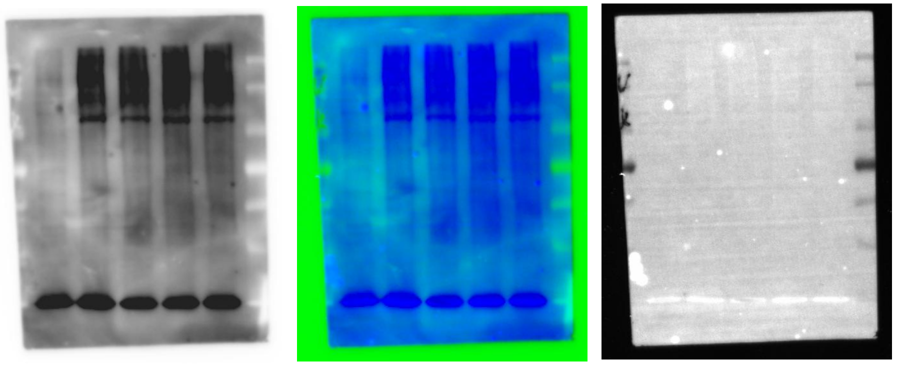

Figure 9. A ubiquitination

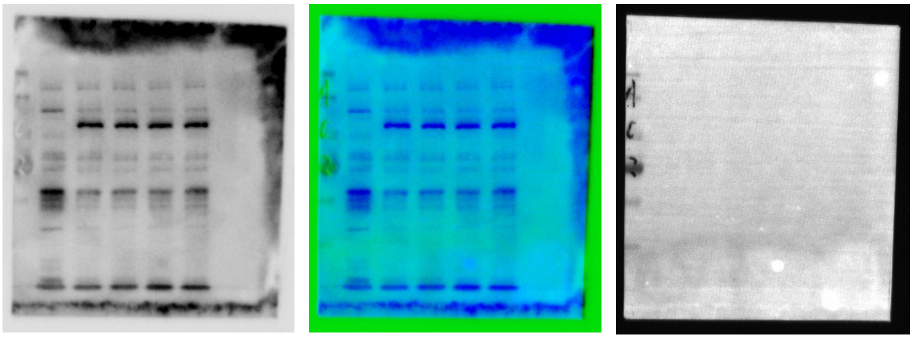

Figure 9. A acetylation

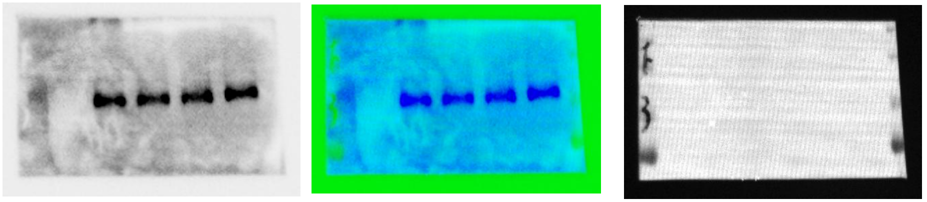

Figure 9. A Flag

Figure 9. A Immunoblotting analysis of the ubiquitination and acetylation levels of STAT1-Flag in the HEK293T cells.

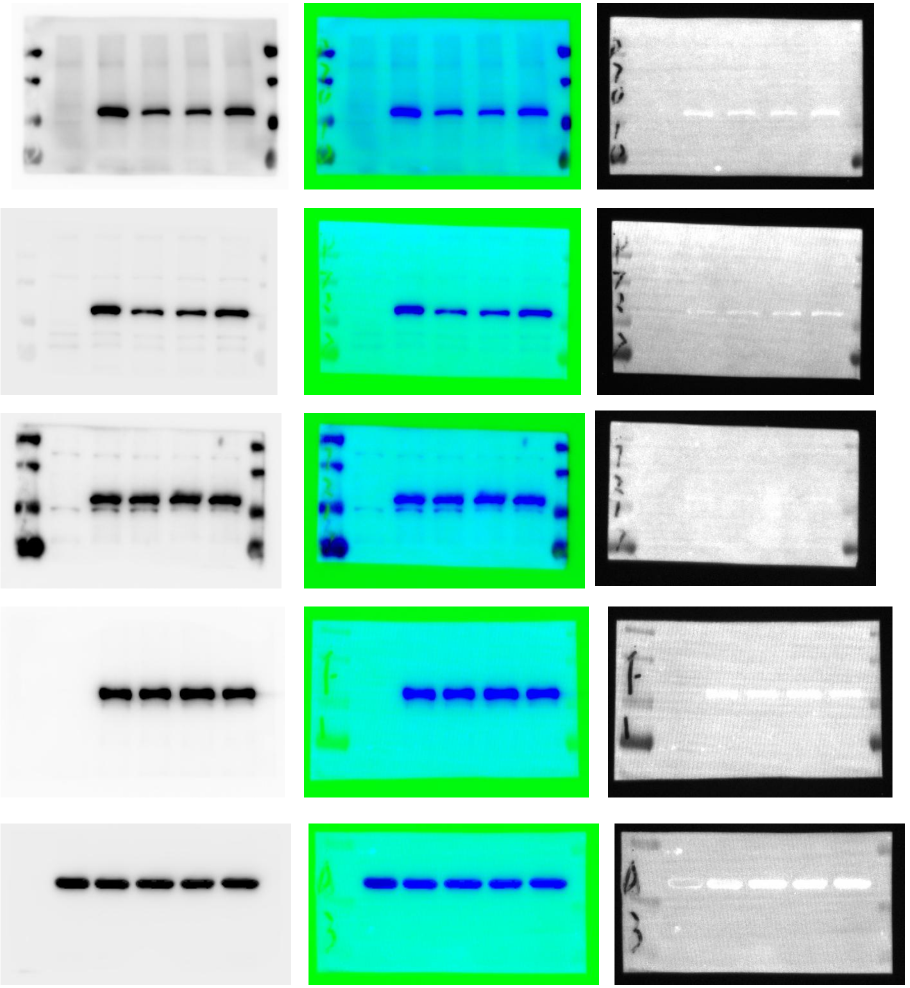

Figure 9. B P-STAT1 701

Figure 9. B P-STAT1 727

Figure 9. B STAT1

Figure 9. B Flag

Figure 9. B actin

Figure 9. B Immunoblotting analysis of P-STAT1 expression in the HEK293T cells

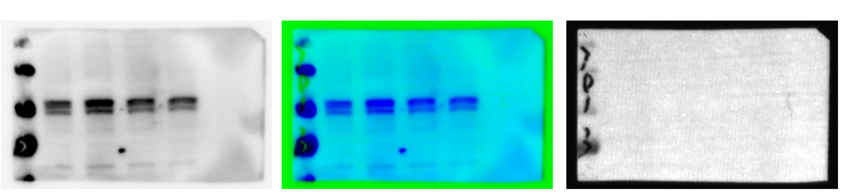

Figure 9. C P-STAT1 701

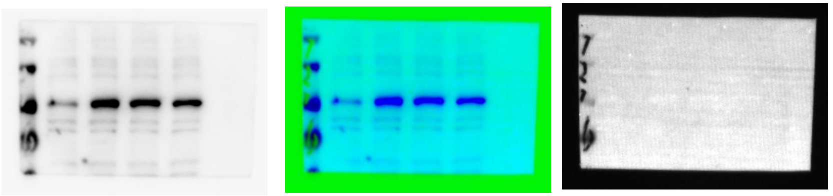

Figure 9. C P-STAT1 727

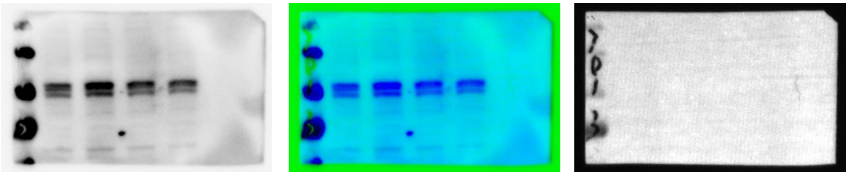

Figure 9. C STAT1

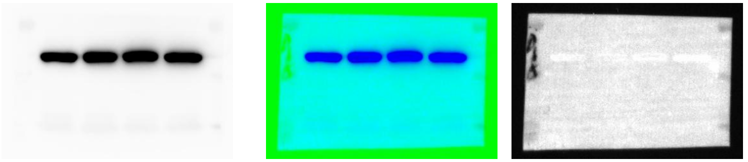

Figure 9. C actin

Figure 9. C Immunoblotting analysis of P-STAT1 expression in the RAW264.7 cells

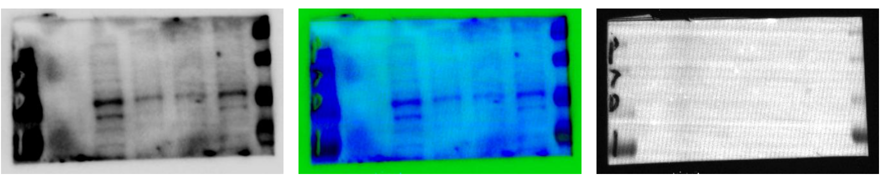

Figure 9. F P-STAT1 701

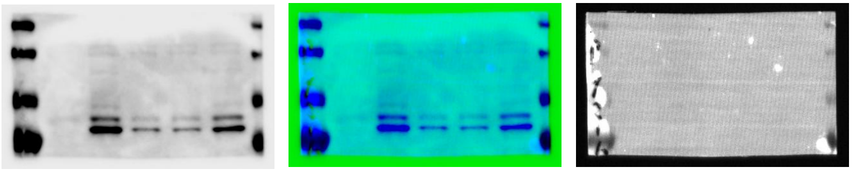

Figure 9. F P-STAT1 727

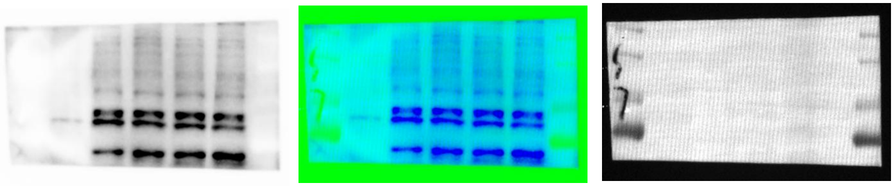

Figure 9. F STAT1

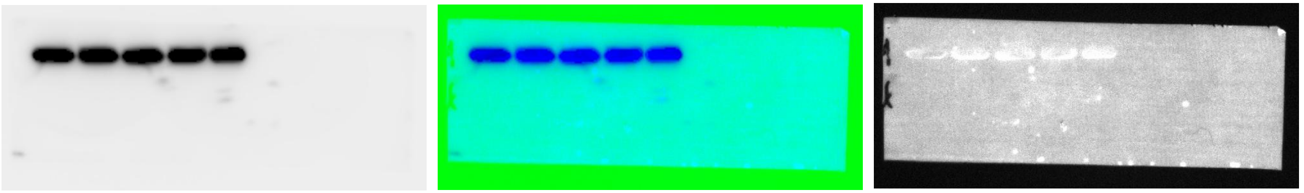

Figure 9. F actin

Figure 9. F Immunoblotting analysis of P-STAT1 expression in the STAT1-KO RAW264.7 cells

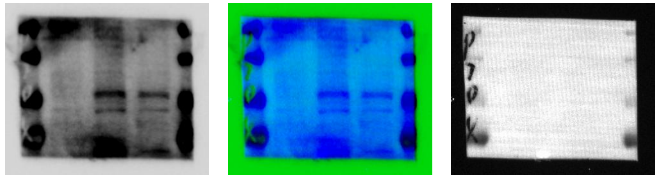

Figure 9. J P-STAT1 701

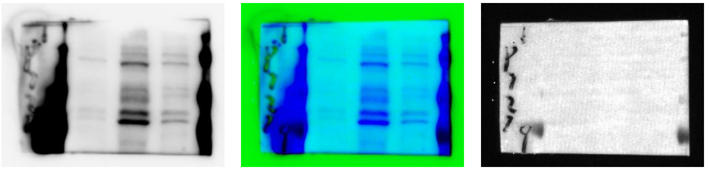

Figure 9. J P-STAT1 727

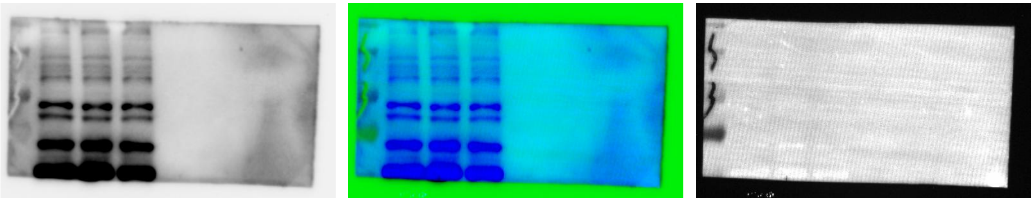

Figure 9. J STAT1

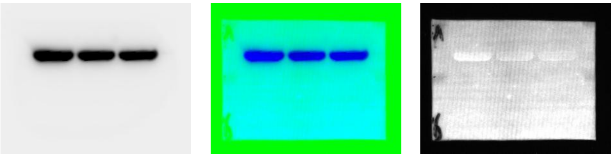

Figure 9. J actin

Figure 9. J Immunoblotting analysis of P-STAT1 expression in the STAT1-KO RAW264.7 cells

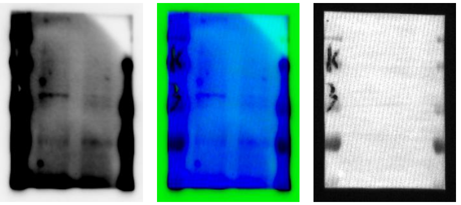

Figure 10. F Kbhb

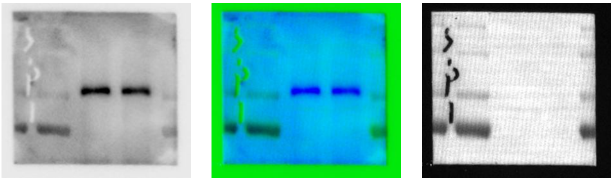

Figure 10. F STAT1

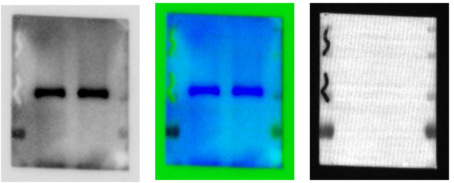

Figure 10. F STAT1

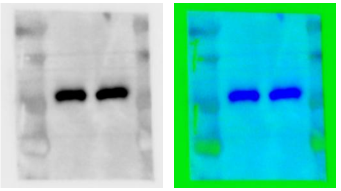

Figure 10. F Flag

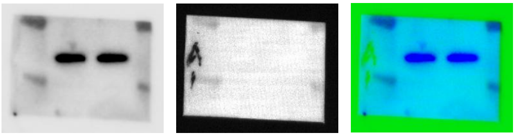

Figure 10. F actin

Figure 10. F Immunoblotting analysis of STAT1 Kbhb levels in mouse PMs

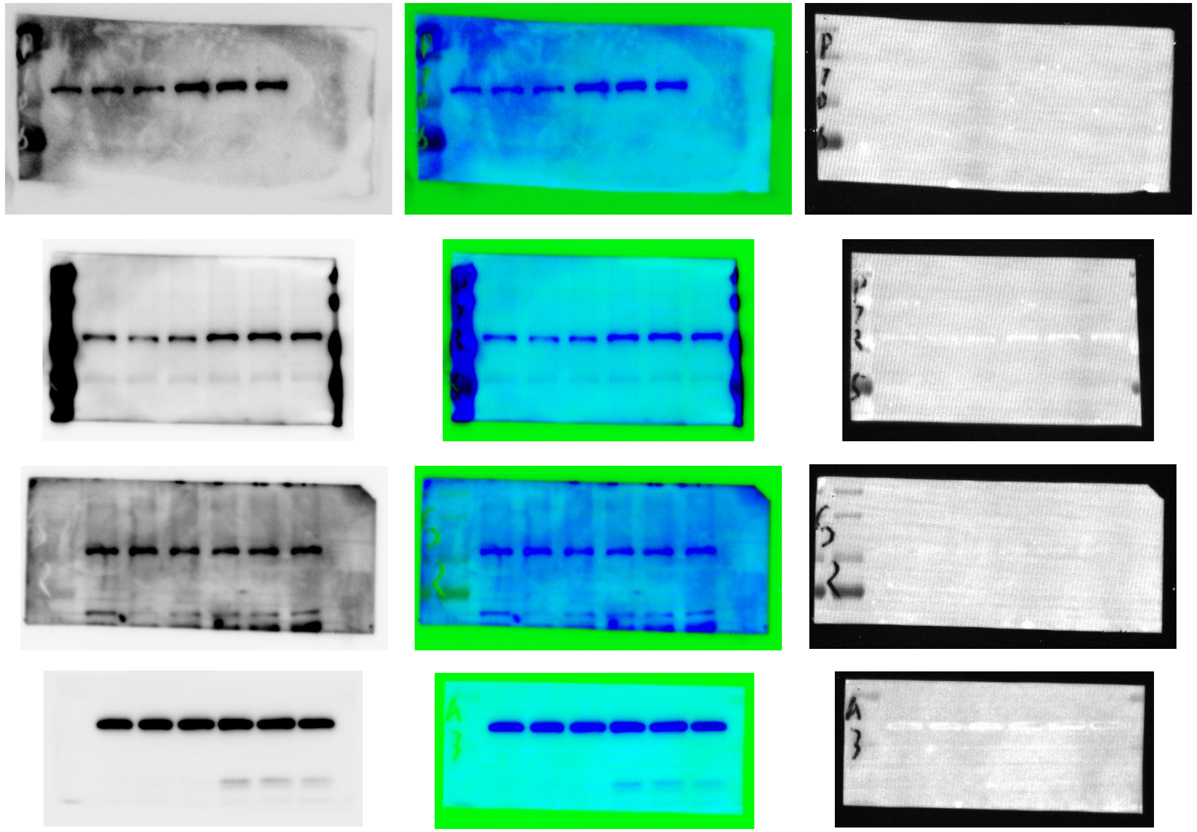

Figure 10. G P-STAT1 701

Figure 10. G P-STAT1 727

Figure 10. G STAT1

Figure 10. G actin

Figure 10. F Immunoblotting analysis of P-STAT1 expression in mouse PMs

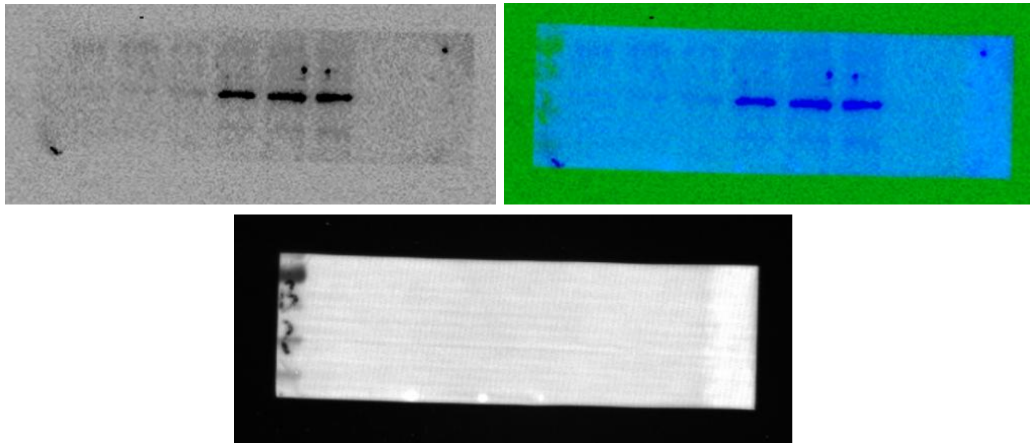

S Figure 2. A BDH1

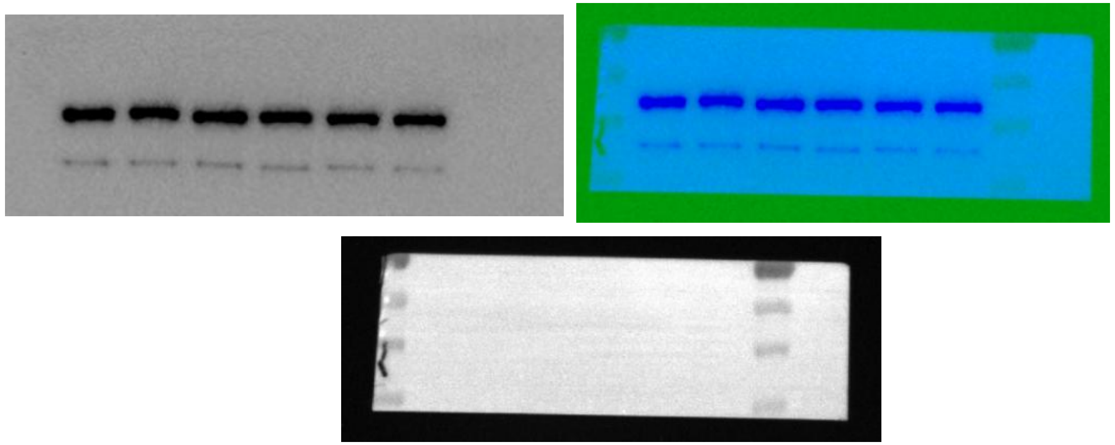

S Figure 2. A actin

S Fig. 2 A Immunoblotting results confirmed the successful overexpression of BDH1.

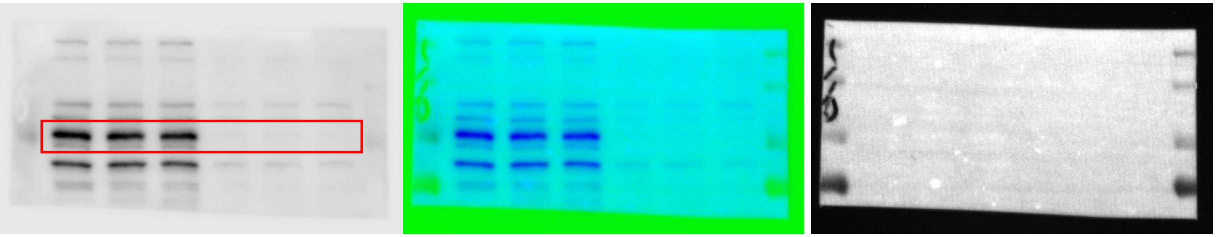

S Figure 4. B STAT1

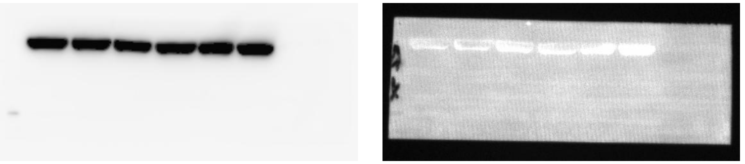

S Figure 4. B actin

S Fig. 4 B Immunoblotting results confirmed the successful KO of STAT1.
